# ﻿A consolidated phylogeny of snail-eating snakes (Serpentes, Dipsadini), with the description of five new species from Colombia, Ecuador, and Panama

**DOI:** 10.3897/zookeys.1143.93601

**Published:** 2023-01-25

**Authors:** Alejandro Arteaga, Abel Batista

**Affiliations:** 1 Biodiversity Field Lab (BioFL), Khamai Foundation, Quito, Ecuador Biodiversity Field Lab Quito Ecuador; 2 Tropical Herping S.A., Quito, Ecuador Tropical Herping S.A. Quito Ecuador; 3 Universidad Autónoma de Chiriquí (UNACHI), Vicerrectoría de investigación y Postgrado, David, Chiriquí, Panama Universidad Autónoma de Chiriquí David Panama; 4 Museo Herpetológico de Chiriquí (MHCH), David, Chiriquí, Panama Museo Herpetológico de Chiriquí David Panama; 5 Fundación Los Naturalistas, Boquete, Chiriquí, Panama Fundación Los Naturalistas Boquete Panama; 6 Sistema Nacional de Investigación (SNI), SENACYT, Panama Sistema Nacional de Investigación SENACYT Panama

**Keywords:** Caenophidia, Colubroidea, *Dipsas*, *Plesiodipsas*, *Sibon*, Squamata, systematics, taxonomy

## Abstract

A molecular phylogeny of the Neotropical snail-eating snakes (tribe Dipsadini Bonaparte, 1838) is presented that includes 60 of the 133 species currently recognized. There is morphological and phylogenetic support for four new species of *Sibon* Fitzinger, 1826 and one of *Dipsas* Laurenti, 1768, which are described here based on their unique combination of molecular, meristic, and color pattern characteristics. *Plesiodipsas*Harvey et al., 2008 is designated as a junior synonym of *Dipsas* and additional evidence is presented to support the transfer of the genus *Geophis* Wagler, 1830 to the tribe Dipsadini. Two of the subspecies of *S.nebulatus* (Linnaeus, 1758) are elevated to full species status. Insight into additional undescribed cryptic diversity within the *S.nebulatus* species complex is provided. Evidence that supports the existence of an undescribed species previously confused with *D.temporalis* is provided, as well as the first country record of *S.ayerbeorum* Vera-Pérez, 2019 in Ecuador with a comment on the ontogenetic variation of the latter. Finally, photographs of Colombian, Ecuadorian, and Panamanian snail-eating snakes are provided.

## ﻿Introduction

The snail-eating snake tribe Dipsadini is one of the most diverse yet taxonomically complex group of snakes in the Neotropics. Many authors (Peters 1960; Downs 1961; Hoge 1964; Peters and Orejas-Miranda 1970; Kofron 1982; Orcés and Almendáriz 1987; Porto and Fernandes 1996; Fernandes et al. 1998, 2002; Cadle and Myers 2003; Passos et al. 2004, 2005; Cadle 2005, 2007; Harvey 2008; Harvey and Embert 2008; Harvey et al. 2008) have attempted to clarify the systematics of the group or its subgroups using morphological characters. However, the majority of these authors disagree about the number of genera included in the tribe as well as the allocation of species among genera. Fortunately, this lack of consensus is likely coming to an end with the use of molecular tools on Dipsadini systematics. Three independent groups of researchers (Sheehy 2012; Arteaga et al. 2018; and Grünwald et al. 2021) arrived at similar conclusions about the contents and limits of this snake tribe when molecular and morphological evidence were combined. Although progress is being made regarding the taxonomy of Dipsadini, more than half of the species of the group remain unsampled for DNA characters and additional diversity remains undescribed.

In his unpublished PhD thesis, Sheehy (2012) presented a large (194 taxa) phylogeny of the group using two mitochondrial ant two nuclear genes. Among his most important findings were that the genus *Sibynomorphus* Fitzinger, 1843 is paraphyletic with respect to *Dipsas* and that the genus *Geophis* Wagler, 1830 is deeply nested within Dipsadini. He also uncovered two major (one South American and one Central American) and eleven minor geographically structured clades within the widely distributed snake species *Sibonnebulatus* and found that *Sibonannulatus* (Günther, 1872) is paraphyletic with respect to *Sibonlamari* Solórzano, 2001 and *Sibonperissostichon*Köhler et al., 2010. Sheehy (2012) also presented phylogenetic evidence that *Geophissanniolus* (Cope, 1866) (previously in the genus *Sibon*) and *D.gaigeae* (Oliver, 1937) do not belong to their nominal genera and their relationships with the remaining groups of snail-eating snakes are ambiguous. Since Sheehy’s work was not published, his findings were not integrated into the taxonomy of the Dipsadini. However, his work provided a solid framework for comparison for two subsequent studies.

First, Arteaga et al. (2018) presented a phylogeny of the group based on novel taxon sampling. This work differed from Sheehy’s in that it had an emphasis on *Dipsas* (rather than *Sibon*) and on South American (rather than Central American) Dipsadini in general. However, it confirmed some of the results of Sheehy (2012); most notably the paraphyly of *Dipsas* with respect to *Sibynomorphus*, which resulted in the latter being designated as a junior subjective synonym of *Dipsas*. Besides describing five new species of Dipsadini, Arteaga et al. (2018) uncovered, but did not explore further, high levels of intraspecific divergence within each of the nominal species *D.vermiculata* Peters, 1960 and *Sibonannulatus*, which suggested that further diversity remained to be described in this group. They also presented evidence that *Sibonnebulatus* is paraphyletic with respect to *Sibondunni* Peters, 1957 and *Sibonbevridgelyi*Arteaga et al., 2018, and recognized that elevating the subspecies *Sibonnebulatusleucomelas* (Boulenger, 1896) and *Sibonnebulatushartwegi* Peters, 1960 to full species status would help resolve this paraphyly. However, Arteaga et al. (2018) refrained from proposing further taxonomic arrangements because their sample size for *D.vermiculata*, *Sibonannulatus*, and *Sibonnebulatus* was insufficient. Later, Grünwald et al. (2021) combined some of the DNA sequences of Sheehy (2012) with novel sequences from Mexico into a phylogeny of Dipsadini that focused on the genus *Tropidodipsas* Günther, 1858. Based on the results of their phylogenetic analyses, these authors transferred *T.annuliferus* (Boulenger, 1894), *T.sartorii* (Cope, 1863), and *Sibonsanniolus* to *Geophis*, a genus that, according to Sheehy (2012), should be added to the tribe Dipsadini, a decision seconded by Grünwald et al. (2021) and also herein. Lastly, in a project seeking to create a large DNA barcode library of reptiles from the National Museum of Natural History tissue holdings, Mulcahy et al. (2022) provided mitochondrial DNA sequences (gene fragments COI and 16S) for ten species of Dipsadini. However, these have not been included in any phylogenetic studies so far.

Here, we combine the datasets of Sheehy (2012), Arteaga et al. (2018), Grünwald et al. (2021), and Mulcahy et al. (2022) with novel DNA sequences of Colombian, Panamanian, and Ecuadorian material into a consolidated phylogeny of the tribe Dipsadini. Notably, we include the recently described *Sibonayerbeorum* and the monotypic *Plesiodipsasperijanensis* (Aleman, 1953) in the analysis. The combined molecular sampling, together with morphological analysis and species distribution models, supports the existence of at least five new species of Neotropical snail-eating snakes, which we describe here.

## ﻿Materials and methods

### ﻿Ethics statement

This study was carried out in strict accordance with the guidelines for use of live amphibians and reptiles in field research (Beaupre et al. 2004) compiled by the American Society of Ichthyologists and Herpetologists (**ASIH**), the Herpetologists’ League (**HL**) and the Society for the Study of Amphibians and Reptiles (**SSAR**). All procedures with animals (see below) were reviewed by the Ministerio del Ambiente, Agua y Transición Ecológica (**MAATE**), Ecuador and UNARGEN-Ministerio de Ambiente Panamá, and specifically approved as part of obtaining the following field permits for research and collection: MAE-DNB-CM-2018-0105 and MAATE-DBI-CM-2022-0245 (granted to Universidad San Francisco de Quito) and SC/A-8-09, SC/A-28-09, SC/A-37-11, SC/A-33-12, SE/A-60-16, and SE/A-33-18 (granted to Museo Herpetológico de Chiriquí). Specimens were euthanized with 20% benzocaine, fixed in 10% formalin or 90% ethanol, and stored in 70% ethanol. Museum vouchers were deposited at Museo de Zoología de la Universidad San Francisco de Quito (**ZSFQ**), Museo Herpetológico de Chiriquí (**MHCH**), and at the Senckenberg Forschungsinstitut Frankfurt (**SMF**). Specimens labeled TH, SC, and JMG were also deposited at ZSFQ.

### ﻿Common names

Criteria for common name designation are as proposed by Caramaschi et al. (2006) and Coloma and Guayasamin (2011–2017), reviewed by Arteaga et al. (2019). These are as follows (in order of importance): (i) the etymological intention (implicit or explicit) that the authors used when naming the species (specific epithet); (ii) a common name that is already widely used in the scientific literature; (iii) a common name that has an important ancestral or cultural meaning; (iv) a common name based on any distinctive aspect of the species (distribution, morphology, behavior, etc).

### ﻿Morphological data

Our terminology for Dipsadini cephalic shields follows proposals by Peters (1960) and Harvey and Embert (2008). Diagnoses and descriptions generally follow Fernandes et al. (2010) and ventral and subcaudal counts follow Dowling (1951). We physically examined comparative alcohol-preserved specimens from the herpetology collections at Colección de Prácticas Zoológicas de la Universidad del Valle (**CPZ-UV**), Colección Zoológica de la Universidad ICESI (**CZI**), División de Herpetología del Instituto Nacional de Biodiversidad (**DHMECN**), MHCH, Museum d’Histoire Naturelle de la Ville de Genève (**MHNG**), Museo de Zoología de la Universidad del Azuay (**MZUA**), Museo de Zoología de la Universidad Tecnológica Indoamérica (**MZUTI**), SMF, Colección de Anfibios y Reptiles de la Universidad del Valle (**UV-C**), and ZSFQ (Suppl. material 1). We also examined photographs of specimens housed at Museo de Zoología de la Pontificia Universidad Católica del Ecuador (**QCAZ**). Morphological measurements were taken with measuring tapes to the nearest 1 mm, or with digital calipers to the nearest 0.1 mm. Abbreviations are as follows: snout-vent length (**SVL**); tail length (**TL**); total length, **TOL** (SVL + TL). Sex was determined by establishing the presence/absence of hemipenes through a subcaudal incision at the base of the tail unless hemipenes were everted.

### ﻿Sampling

Tissue samples from 19 individuals representing eight species (including the five new species described here) were obtained in Colombia, Ecuador, and Panama. All specimens included in the genetic analyses were morphologically identified according to Peters (1960), Duellman (1978), Savage (2002), Cadle and Myers (2003), Cadle (2005, 2007), Harvey (2008), Harvey and Embert (2008), and Arteaga et al. (2018). We generated sequence data for samples marked with an asterisk in Appendix 1, which includes museum vouchers from MHCH, MZUTI, SMF, Colección de Herpetología de la Universidad Industrial de Santander (**UIS**), and ZSFQ.

### ﻿Laboratory techniques

Genomic DNA was extracted from 96% ethanol-preserved tissue samples (liver, muscle tissue, or scales) using either a guanidinium isothiocyanate extraction protocol (Peñafiel et al. 2020), or a modified salt precipitation method based on the Puregene DNA purification kit (Gentra Systems). The nucleotide sequences of the primers and the PCR conditions applied to each primer pair are detailed in Appendix 2. PCR products were cleaned with either ExoSAP-IT (Affymetrix, Cleveland, OH), or Exonuclease I and Alkaline Phosphatase (Illustra ExoProStar by GE Healthcare) before they were sent to Macrogen Inc (Seoul, South Korea) for sequencing. All PCR products were sequenced in both forward and reverse directions with the same primers that were used for amplification. The edited sequences were deposited in GenBank (Appendix 1).

### ﻿DNA phylogenetic analyses

A total of 343 DNA sequences was used to build a phylogenetic tree of the tribe Dipsadini, of which 35 were generated during this work and 308 were downloaded from GenBank, most of which were produced by Sheehy (2012), Arteaga et al. (2018), and Grünwald et al. (2021). Of these, 20 sequences are 242–473 bp long fragments of the 12S gene, 65 are 201–422 bp long fragments of the 16S gene, 16 are 493–657 bp long fragments of the COI gene, 85 are 559–1,071 bp long fragments of the CYTB gene, 80 are 325–684 bp long fragments of the ND4 gene, 29 are 606–674 bp long fragments of the DNAH3 gene, and 48 are 456–470 bp long fragments of the NT3 gene. New sequences were edited and assembled using the program Geneious ProTM 2021.1.1 (Drummond et al. 2021) and aligned with those downloaded from GenBank (Appendix 1) using MAFFT v. 7 (Katoh and Standley 2013) under the default parameters in Geneious ProTM 2021.1.1. Genes were combined into a single matrix with 17 partitions, one per non-coding gene and three per protein coding gene corresponding to each codon position. The best partition strategies along with the best-fit models of evolution were obtained in PartitionFinder 2.1.1 (Lanfear et al. 2016) under the Bayesian information criterion.

Phylogenetic relationships were assessed under a Bayesian inference (BI) approach in MrBayes 3.2.0 (Ronquist and Huelsenbeck 2013). Four independent analyses were performed to reduce the chance of converging on a local optimum. Each analysis consisted of 20,000,000 generations and four Markov chains with default heating settings. Trees were sampled every 1,000 generations and 25% of them were arbitrarily discarded as ‘‘burn-in.” The resulting 15,000 saved trees per analysis were used to calculate posterior probabilities (PP) for each bipartition in a 50% majority-rule consensus tree. We used Tracer 1.6 (Rambaut et al. 2022) to assess convergence and effective sample sizes (ESS) for all parameters. Additionally, we verified that the average standard deviation of split frequencies between chains and the potential scale reduction factor (PSRF) of all the estimated parameters approached values of ≤ 0.01 and 1, respectively. GenBank accession numbers are listed in Appendix 1.

### ﻿Distribution maps and ecological niche models

We present ranges of occurrence for eleven species of Dipsadini, including five new species described here. Presence localities are derived from museum vouchers (Suppl. materal 1), photographic records (iNaturalist), and the literature (all summarized in Suppl. materal 2). For each species, a binary environmental niche model (ENM) accompanies the dot maps. These models estimate potential areas of distribution on the basis of observed presences and a set of environmental predictors (Elith and Leathwick 2009). To delimit the occupancy areas and the potential species distribution, we used the BAM diagram proposal (Soberón and Peterson 2005; Peterson et al. 2011). To create the models, we used presence localities listed in Suppl. materal 2, 19 bioclimatic variables from Worldclim 1.4 (Hijmans et al. 2005), and Maxent 3.4.1k, an algorithm based on the principle of maximum entropy (Phillips et al. 2006; Elith et al. 2011; Renner and Warton 2013).

For the first explorative exercise, we used the 19 climate layers from the WorldClim project and assessed which variables were the most important for the model, according to the Jackknife test calculated in MaxEnt (Royle et al. 2012). Correlated environmental variables (r < 0.8) were identified using the PEARSON correlation test of PAST 3. In a second modelling exercise, we used the locality records for each species and the variables identified in the first approach to generate the species distribution. 5,000 iterations were specified to the program with clamping and no extrapolation. All other parameters in MaxEnt were maintained at default settings. To create the binary environmental niche models, suitable areas were distinguished from unsuitable areas by setting a minimum training presence threshold value. The logistic format was used to obtain the values for habitat suitability (continuous probability from 0 to 1), which were subsequently converted to binary presence-absence values on the basis of the established threshold value, defined herein as the minimum training presence. The convergence threshold was set to 10^-5^, maximum iterations to 500, and the regularization parameter to “auto.”

## ﻿Results

### ﻿Molecular phylogeny and taxonomic consequences

**Figure 1.**
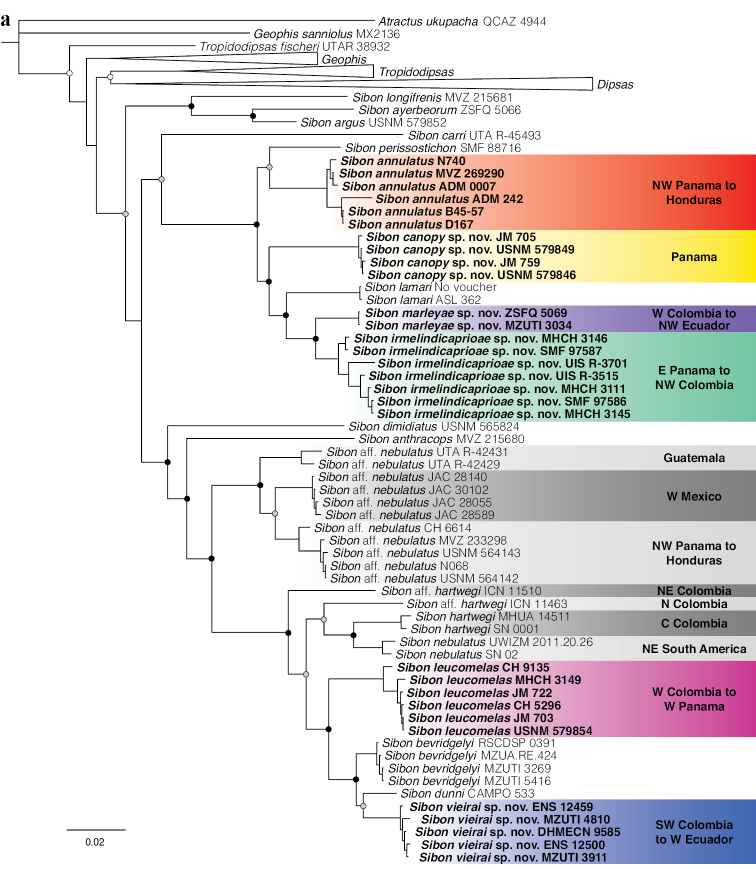
Phylogenetic relationships within Dipsadini inferred using a Bayesian inference and derived from analysis of DNA gene fragments 12S, 16S, COI, CYTB, ND4, DNAH3, and NT3. Support values on intra-specific branches are not shown for clarity. Voucher numbers for sequences are indicated for each terminal. Black dots indicate clades with posterior probability values from 95–100%. Grey dots indicate values from 70–94%. White dots indicate values from 50–69% (values < 50% not shown). Colored clades correspond to the species’ distribution presented in the maps. New or redefined species are indicated in bold type.

Selected partitions and models of evolution are presented in Table 1. We consider strong support for a clade when Bayesian analyses yield posterior probability values > 95%, following Felsenstein (2004). The topology and support (Fig. 1) of our phylogenetic tree differs from that of Sheehy (2012), Arteaga et al. (2018), and Grünwald et al. (2021), primarily regarding the relationships between the included genera. Below, we outline these differences and comment on the phylogenetic position of new material included in this work.

**Table 1.** Partition scheme and models of evolution used in phylogenetic analyses. Numbers in parentheses indicate codon position.

Partitition	Best model	Gene regions	Number of aligned sites
1	GTR+I+G	12S, 16S, COI(1), CYTB(3), ND4(1)	1742
2	HKY+I+G	CYTB(1), ND4(2)	614
3	GTR+I+G	COI(3), CYTB(2), ND4(3)	833
4	K80+I	DNAH3(1), DNAH3(2)	450
5	K80+G	DNAH3(3), NT3(1)	381
6	K80+G	NT3(2), NT3(3)	313
7	F81	COI(2)	219

*Tropidodipsasfischeri* Boulenger, 1894 is recovered as sister to all other sampled Dipsadini, with the exception of *Geophissanniolus*, a species that did not form a group with the remaining *Geophis*. Neither of these or any other higher relationships within Dipsadini are strongly supported in our analysis, but all other sampled species of Dipsadini are included in their corresponding genera *sensu*Arteaga et al. (2018) and Grünwald et al. (2021).

With the exception of *Geophissanniolus*, relationships within *Geophis* are identical to those presented in Grünwald et al. (2021). The relationships within *Tropidodipsas* are similar to those presented in Sheehy (2012) and the newly described *T.tricolor*Grünwald et al., 2021 is recovered as the strongly supported sister species of *T.papavericola*Grünwald et al., 2021.

*Dipsasgaigeae* is recovered as the sister species of all other *Dipsas*, albeit with low support, but not as sister to (see Sheehy 2012) or forming a polytomy with (see Grünwald et al. 2021) *Tropidodipsas*. The *D.articulata* group, as defined by Peters (1960) and modified by Harvey (2008) and Arteaga et al. (2018) is monophyletic. Within it, *D.viguieri* (Bocourt, 1884), a species not included in previous phylogenetic analyses, is recovered as the moderately supported sister taxon of *D.articulata* (Cope, 1868). We found *D.pavonina* Schlegel, 1837 to be the moderately supported sister taxon of a clade formed by *D.peruana* (Boettger, 1898), *D.palmeri* (Boulenger, 1912), and *D.klebbai*Arteaga et al., 2018, a relationship not recovered in any of the previous phylogenies. *Plesiodipsasperijanensis* is nested within the genus *Dipsas* and is recovered as the moderately supported sister species of *D.albifrons* (Sauvage, 1884). There are two reciprocally monophyletic, deeply divergent, and geographically structured clades within *D.vermiculata* sensu lato. One is *D.vermiculata* sensu stricto and the other is a new species endemic to the Cordillera del Cóndor in southeastern Ecuador and northern Peru. This new species is described in this work. The *D.oreas* group, as defined by Harvey (2008) and modified by Arteaga et al. (2018) is monophyletic and includes *D.nicholsi* Dunn, 1933. Within it, *D.elegans* Boulenger, 1896 is recovered as the strongly supported sister species of *D.ellipsifera* (Boulenger, 1898), a relationship already uncovered in Arteaga et al. (2018). There are two reciprocally monophyletic, deeply divergent, and geographically structured clades within *D.temporalis* (Werner, 1909). One is *D.temporalis* sensu stricto and the other is a new species endemic to central Panama.

Relationships within *Sibon* are most similar to those presented in Sheehy (2012). The *S.argus* group is sister to all other members of the genus and it includes the newly described *S.ayerbeorum*, a species not previously sampled for molecular characters. *Sibonannulatus* is paraphyletic with respect to *S.perissostichon*, *S.lamari*, and three new species described in this work. We restrict the name *S.annulatus* to the red clade in Fig. 1a based on the type locality of this species (Cartago, Costa Rica) where only members of the red clade have been recorded, as well as on the original description of this species. Günther (1872) mentioned that the holotype has 164 ventrals and the body and tail are encircled by black rings. Members of the yellow clade have more than 170 ventrals and lack full body rings. *Sibonannulatus*, *S.perissostichon*, *S.lamari*, and the three new species form a monophyletic unit exclusive of all other species of the paraphyletic *S.annulatus* species group (see Arteaga et al. 2018 for a list of species included in this group). There are eleven monophyletic, deeply divergent, and geographically structured clades within *S.nebulatus* sensu lato. Two of these correspond to species already described (*S.bevridgelyi* and *S.dunni*), three correspond to subspecies of *S.nebulatus* (*nebulatus*, *leucomelas*, and *hartwegi*), one corresponds to a new species described here, and the remaining clades are deemed putative new species. The allocation of subspecies names to each clade was based on direct examination of museum vouchers and whether these agree in coloration and lepidosis with the corresponding holotype, as well as on the geographic range of the included samples.

Finally, we excluded *Sibonnoalamina*Lotzkat et al., 2012 (voucher SMF 91539) from the analyses as the short sequence available in GenBank (gene fragment 16S) represented a rogue taxon that assumed varying phylogenetic positions in the tree collection used to build the consensus tree.

## ﻿Systematic accounts

We name and provide descriptions only for species that are monophyletic in our molecular phylogeny and share diagnostic features of their coloration pattern and lepidosis. Based on these species delimitation criteria, which follow the general species concept of de Queiroz (2007), we describe five new species of Dipsadini.

### ﻿ Sibon irmelindicaprioae sp. nov.

Taxon classificationAnimaliaSquamataColubridae

5C3151DE-21DD-56FD-A582-39BBA2799285

https://zoobank.org/E2264D87-D5DF-4977-9893-B85C7F762644

**Figure 2.**
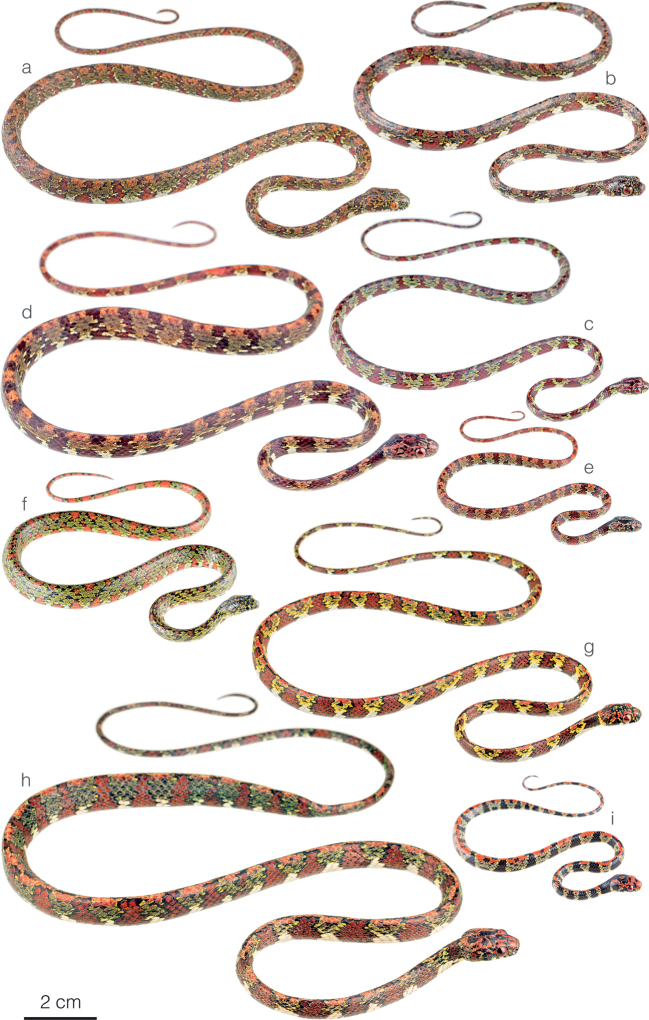
Photographs of some species of *Sibon* in life **a***S.irmelindicaprioae* sp. nov. MHCH 3269 from Chucantí Reserve, Darién province, Panama **b***S.irmelindicaprioae* sp. nov. from Morromico Reserve, Chocó department, Colombia **c***S.canopy* sp. nov. from Cerro Gaital, Coclé province, Panama **d, e***S.annulatus* from Centro Manu, Limón province, Costa Rica **f***S.ayerbeorum*ZSFQ 5066 from Canandé Biological Reserve, Esmeraldas Province, Ecuador **g***S.marleyae* sp. nov. holotype ZSFQ 5065 from Verdecanandé, Esmeraldas Province, Ecuador **h***S.marleyae* sp. nov. ZSFQ 5068 from Verdecanandé, Esmeraldas Province, Ecuador **i***S.marleyae* sp. nov. neonate from Verdecanandé, Esmeraldas Province, Ecuador.

**Figure 3.**
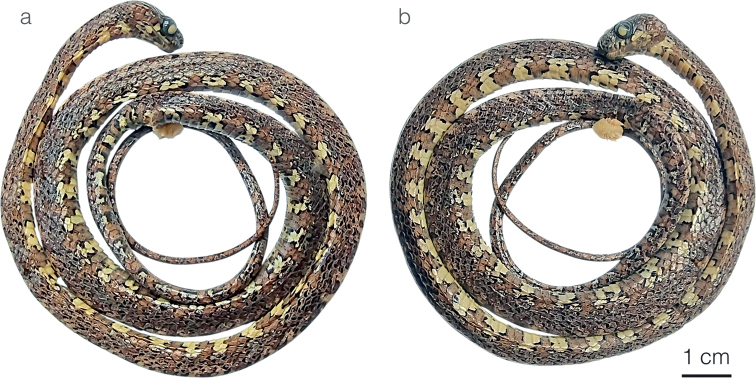
Adult male holotype of *Sibonirmelindicaprioae* sp. nov. MHCH 3143 in lateral views **a** right and **b** left side.

**Figure 4.**
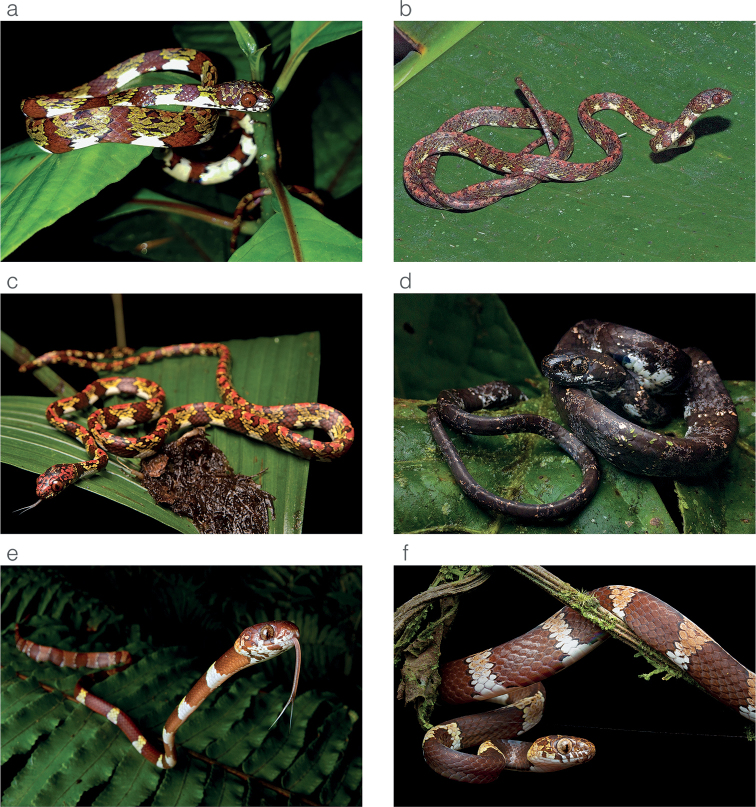
Photographs of some species of *Sibon* and *Dipsas* in life **a***S.canopy* sp. nov. from El Valle de Antón, Coclé province, Panama **b***S.irmelindicaprioae* sp. nov. holotype MHCH 3143 from Puerto Indio, Darién province, Panama **c***S.marleyae* sp. nov. from Verdecanandé, Esmeraldas Province, Ecuador **d***S.vieirai* sp. nov. from Mashpi Amagusa Reserve, Pichincha province, Ecuador **e***Dipsas* sp. from Cerro Gaital, Coclé province, Panama **f***D.welborni* sp. nov. ZSFQ 5060 from Vía a Nuevo Paraíso, Zamora Chinchipe province, Ecuador.

**Figure 5.**
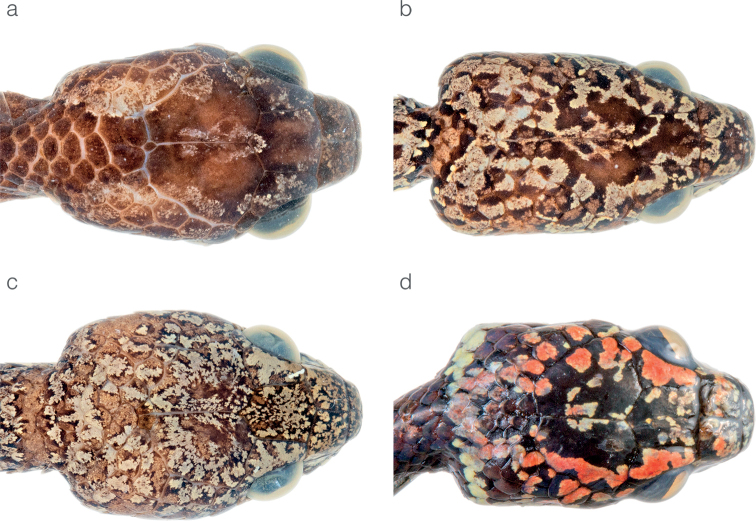
Differences in head morphology between species of *Sibon* previously subsumed under *S.annulatus***a***S.annulatus*MHCH 1982 from Bonyic, Bocas del Toro province, Panama **b***S.canopy* sp. nov. holotype MHCH 3110 from Cerro Gaital, Coclé province, Panama **c***S.irmelindicaprioae* sp. nov. MHCH 3120 from Pirré, Darién province, Panama **d***S.marleyae* sp. nov. holotype ZSFQ 5065 from Verdecanandé, Esmeraldas Province, Ecuador.

Figs 2a, b , 3 , 4b , 5c Proposed standard English name: DiCaprio’s Snail-eating Snake Proposed standard Spanish name: Culebra caracolera de DiCaprio

#### Type material.

***Holotype***: MHCH 3143 (Figs 3, 4b), adult male collected by Abel Batista and Milan Vesely, on 29 September 2011 at Cerro Bailarín, Pavarandó, Comarca Emberá-Wounaan, Panama (7.69385, -78.04267; 852 m a.s.l.).

***Paratypes***: MHCH 3145, adult female collected by Abel Batista and Milan Vesely on 26 September 2012 at Ambroya, Panama province, Panama (8.91680, -78.61779; 484 m a.s.l.). MHCH 3146, adult male collected by Abel Batista on 16 November 2012 at Cerro Garra Garra, Pavarandó, Comarca Emberá-Wounaan, Panama (7.76400, -78.10063; 655 m a.s.l.). MHCH 3111, adult male collected by Abel Batista, Madian Miranda, Orlando Garcés, Rogemif Fuentes on 15 October 2016 at Chucantí, Darién province, Panama (8.79773, -78.46225; 1295 m a.s.l.). MHCH 3120, adult male collected by Abel Batista, Madian Miranda, Michelle Quiroz, Marcos Ponce on 18 June 2015 at Pirré, Darién province, Panama (7.99695, -77.71040; 550 m a.s.l.). COLZOOCH-H 0792, adult male collected by Jhon Tailor Rengifo Mosquera on 6 March 2005 at El Afirmado, Chocó department, Colombia (5.64190, -77.07550; 216 m a.s.l.).

#### Diagnosis.

*Sibonirmelindicaprioae* sp. nov. is placed in the genus *Sibon* based on phylogenetic evidence (Fig. 1a) and on having the penultimate supralabial conspicuously higher than all other supralabials. The species is diagnosed based on the following combination of characters: (1) 15/15/15 smooth dorsals with enlarged vertebral row (1.5× as wide as adjacent rows); (2) loreal and prefrontal in contact with orbit; (3) 7–9 supralabials with, usually, 5^th^ and 6^th^ contacting orbit; (4) 8–10 infralabials with 3^rd^–7^th^ in contact with chinshields, first pair of infralabials not in contact behind symphysial due to presence of postmentals; (5) 187–196 ventrals in males, 174 in the single female; (6) 110–128 divided subcaudals in males, 117 in the single female; (7) dorsal background color olive with maroon lateral body blotches or irregular bands (2–6 dorsal scales long) and a reddish tint along the vertebral line (Figs 2a, b, 4b), ventral surfaces yellowish white with encroachment from the dorsal maroon blotches and with smaller blackish speckles and marks in-between the blotches, dorsal aspect of head variegated with a mixture of pinkish to maroon and pale olive yellow speckles (Fig. 5c), throat yellowish white with brownish blotches and spots, iris pale olive brown to rich dark brown; (8) 292–387 mm SVL in males, 402 mm in the single female; (9) 123–193 mm TL in males, 204 mm in the single female.

#### Comparisons.

*Sibonirmelindicaprioae* sp. nov. is compared to other species of *Sibon* previously subsumed under *S.annulatus* sensu lato (differences summarized in Table 2). From *S.annulatus* sensu stricto, the new species differs in having the dorsal body bands faint and broken along the vertebral line (Figs 2a, b, 4b) and by having a finely variegated pattern on the dorsal surface of the head (Fig. 5c), whereas in *S.annulatus* the dorsal bands reach over all dorsal and lateral surfaces and extend comparably far onto the ventral surfaces (Figs 2d, e) and the head pattern consists of symmetrical broad blotches (Fig. 5a). *Sibonirmelindicaprioae* sp. nov. differs from *S.canopy* sp. nov. by having two (instead of one) postmental scales, a higher number of infralabials (8–10 instead of 6–10), a higher number of ventrals in males (187–196 instead of 180–189), a finely variegated pattern on the dorsal surface of the head (instead of broad irregular blotches; Fig. 5), and by lacking reddish spots enclosed in the dorsal olive interspaces (Figs 2, 4). *Sibonirmelindicaprioae* sp. nov. differs from *S.marleyae* sp. nov. by having a finely variegated pattern on the dorsal surface of the head (instead of having irregular/symmetrical broad blotches; see Fig. 5), distinct dorsal bands (instead of bands usually broken along the vertebral line), and a higher number (over 177) of ventrals in females.

**Table 2.** Differences in coloration, scale counts, and size between *Sibonannulatus*, *S.canopy* sp. nov., *S.irmelindicaprioae* sp. nov., and *S.marleyae* sp. nov. The range of each continuous variable is from our own sample, Peters (1960), Savage and McDiarmid 1992, Lewis et al. (2013), Lotzkat (2014), and Meneses-Pelayo et al. (2016). The numbers in parentheses represent the sample size.

Variable	*Sibonannulatus*		*Siboncanopy* sp. nov.		*Sibonirmelindicaprioae* sp. nov.		*Sibonmarleyae* sp. nov.	
Dorsum pattern	Reddish bands distinct and extending over the entire dorsal and lateral surfaces		Reddish bands distinct and broken along vertebral line in about half of individuals		Reddish bands faint and broken along vertebral line		Reddish bands distinct and usually broken along vertebral line	
Reddish vertebral spots in interspaces	No		Yes		No		No	
Head pattern	Symmetrical broad blotches		Irregular broad blotches		Finely variegated		Irregular/symmetrical broad blotches	
Supralabials	7–8		6–8		7–9		7–8	
Infralabials	7–9		6–8		8–10		8–9	
Postmentals	2		1		2		2 (1 in QCAZ 16974)	
Sex	Males (*n* = 10)	Females (*n* = 15)	Males (*n* = 12)	Females (*n* = 8)	Males (*n* = 7)	Females (*n* = 1)	Males (*n* = 6)	Females (*n* = 5)
Maximum TOL	707 mm	611 mm	648 mm	536 mm	580 mm	606 mm	657 mm	551 mm
Ventrals	170–192	161–186	180–189	170–185	187–196	174	186–204	176–193
Subcaudals	108–135	113–126	113–130	107–124	110–128	117	130–143	109–128

#### Description of holotype.

Adult male, SVL 387 mm, tail length 193 mm (49% SVL); head length 14.3 mm (3.7% SVL) from tip of snout to angle of jaw; head width 9.0 mm (88% head length) taken at broadest point; snout-orbit distance 2.3 mm; head distinct from neck; snout short, blunt in dorsal outline and rounded in profile; rostral 1.8 mm wide, higher than broad; internasals 1.8 mm wide, broader than long; prefrontals 2.3 mm wide, longer than broad, entering orbit; supraocular 3.6 mm long, longer than broad; frontal 3.7 mm long, pentagonal and with an inward-bent anterior border, in contact with prefrontals, supraoculars, and parietals; parietals 5.8 mm long, longer than broad; nasal divided, in contact with first two supralabials, loreal, prefrontal, internasal, and rostral; loreal 1.4 mm long, longer than high, entering the orbit; eye diameter 3.7 mm; pupil semi-elliptical; no preocular; two postoculars; temporals 1+3 on the right side, 2+3 on the left side; eight supralabials with 5^th^ and 6^th^ contacting orbit on the right side, eight supralabials with 5^th^ and 6^th^ contacting orbit on the left side; symphysial precluded from contacting chinshields by a pair of postmentals; ten infralabials, 3^rd^–7^th^ contacting chinshields; two pair of chinshields longer than wide; dorsal scales in 15/15/15 rows, smooth, without apical pits; 193 ventrals; 128 paired subcaudals; cloacal plate single.

#### Natural history.

Specimens of *Sibonirmelindicaprioae* sp. nov. have been found at night foraging on shrubs, trees, and palm fronds 200–300 cm above the ground in old-growth to moderately disturbed evergreen lowland/foothill forests. Snakes of this species are docile and never attempt to bite. When threatened, individuals may hide the head among body coils and produce a musky and distasteful odor.

#### Distribution.

**Figure 6.**
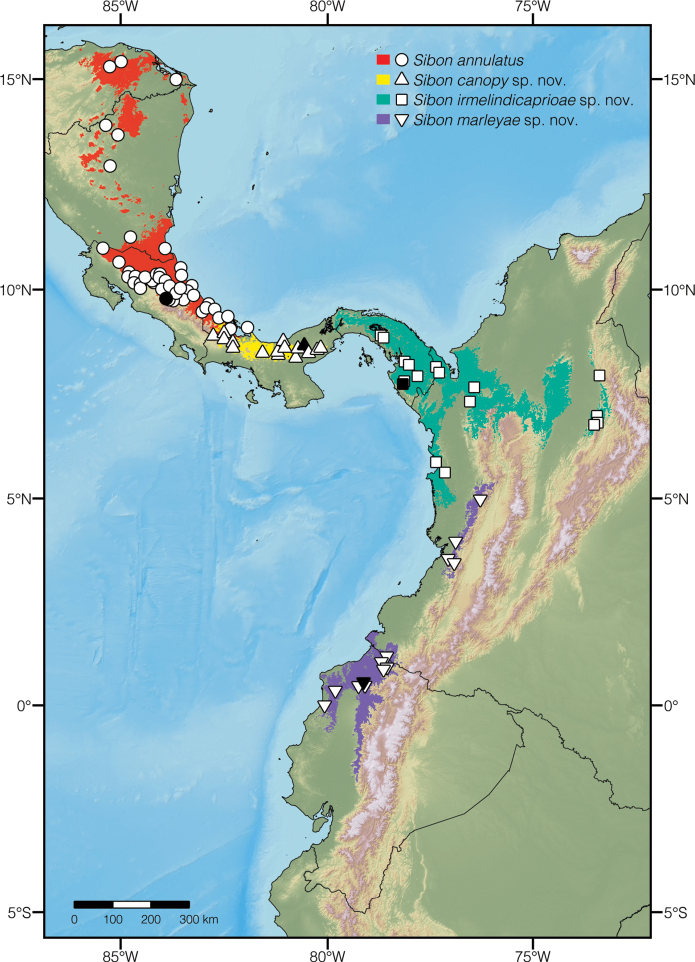
Distribution of species of *Sibon* previously subsumed under *S.annulatus*. Black symbols represent type localities; white symbols other localities listed in Suppl. material 2. Colored areas show the extent of suitable environmental conditions for each species.

*Sibonirmelindicaprioae* sp. nov. is known from 16 localities (listed in Suppl. material 2) in the Chocó region of eastern Panama and northwestern Colombia, with an isolated population on the western slopes of the Cordillera Oriental of Colombia. The species occurs over an estimated area of 62,241 km^2^ and has been recorded at elevations 346–1295 m above sea level (Fig. 6). Since the population on the Cordillera Oriental is isolated from the remaining populations and individuals in this area occur at higher elevations and have a different dorsal color pattern, we provisionally assign them to *S.irmelindicaprioae* sp. nov. pending more comprehensive genetic analyses.

#### Etymology.

The specific epithet *irmelindicaprioae* is a patronym honoring Irmelin DiCaprio (1945–present), mother of Leonardo DiCaprio, long-time advocate and supporter of biodiversity conservation around the world.

#### Conservation status.

We consider *Sibonirmelindicaprioae* sp. nov. to be included in the Near Threatened category following IUCN Red List criteria (IUCN 2001) because the species is distributed over a region that holds large areas of continuous unspoiled forest. Based on the species distribution model presented in Fig. 6 in combination with maps of vegetation cover of Colombia (IDEAM 2014) and Panama (CATHALAC 2011), we estimate that more than half (~ 54%) of the species’ forest habitat is still standing. Unfortunately, vast areas of the Chocó rainforest in northern Colombia and towards central Panama have already been converted to pastures (Myers et al. 2000). However, *S.irmelindicaprioae* sp. nov. occurs over an area greater than 50,000 km^2^ and is presumably not declining fast enough to qualify for a threatened category.

### ﻿ Sibon canopy sp. nov.

Taxon classificationAnimaliaSquamataColubridae

54069384-456A-59F9-A534-BD403B5C43F8

https://zoobank.org/EAE5090E-93AC-403D-A11C-4AFF2C737AE4

**Figure 7.**
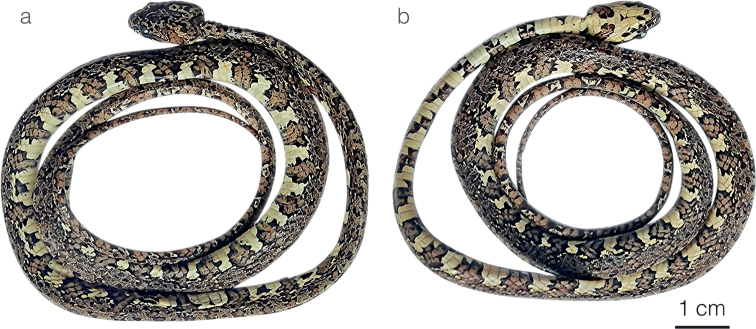
Adult female holotype of *Siboncanopy* sp. nov. MHCH 3110 in **a** dorsal and **b** ventral view.

Figs 2c , 4a , 5b , 7 Proposed standard English name: Canopy Snail-eating Snake Proposed standard Spanish name: Culebra caracolera de dosel

#### Type material.

***Holotype***: MHCH 3110 (Figs 5b, 7), adult female collected by Abel Batista on 8 August 2016 at Cerro Gaital, La Pintada, Coclé province, Panama (8.70874, -80.42411; 543 m a.s.l.).

***Paratypes***: MHCH 1067, SMF 88713–14, juveniles collected by Johannes Köhler, Abel Batista, and Marcos Ponce on 17 January 2007 at Casa de Ancón, Sendero el Pianista, Bocas del Toro province, Panama (8.87142, -82.41594; 1005 m a.s.l.). MHCH 220, juvenile female collected by Abel Batista and Marcos Ponce on March 2002 at Camino al Río Culebra, Bocas del Toro province, Panama (8.90772, -82.39115; 698 m a.s.l.). SMF 85077, adult female collected by Gunther Köhler, Abel Batista, Marcos Ponce, and Javier Sunyer on 17 January 2006 at Reserva Forestal La Fortuna, Comarca Ngäbe-Buglé, Panama (8.77763, -82.20916; 1030 m a.s.l.). SMF 89596, adult female collected by Leonhard Stadler and Nadim Hamad on 5 August 2008 at Cerro Mariposa, Veraguas province, Panama (8.52488, -81.13275; 679 m a.s.l.). SMF 90023, adult female collected by Arcadio Carrizo on 27 June 2008 at Cerro Negro, Veraguas province, Panama (8.56901, -81.09894; 680 m a.s.l.). SMF 91578, adult female collected by Sebastian Lotzkat and Andreas Hertz on 17 July 2010 at Río Changena, Bocas del Toro province, Panama (8.97851, -82.69005; 1641 m a.s.l.). SMF 86411, juvenile collected by Abel Batista and Marcos Ponce on 10 February 2006 at Sendero El Pianista, Bocas del Toro province, Panama (8.87141, -82.41594; 1005 m a.s.l.). SMF 90208, juvenile collected by Joe-Felix Bienentreu and Frank Hauenschild on 25 October 2009 at Cerro Guayabo, Chiriquí province, Panama (8.75531, -82.25431; 1247 m a.s.l.). MHCH 2363–64, males collected by Sebastian Lotzkat and Andreas Hertz between 29 October 2009 and 11 June 2010 at Cabeceras del Río Chiriquí Mali, Comarca Ngäbe-Buglé, Panama (8.78906, -82.21547; 1080 m a.s.l.). MHCH 2365, juvenile male collected by Sebastian Lotzkat and Andreas Hertz on 7 August 2010 at Cerro Mariposa, Veraguas province, Panama (8.50815, -81.12104; 899 m a.s.l.). SMF 85078, adult male collected by Gunther Köhler, Abel Batista, Marcos Ponce, and Javier Sunyer on 19 January 2006 at Reserva Forestal La Fortuna, Comarca Ngäbe-Buglé, Panama (8.77763, -82.20916; 1030 m a.s.l.). SMF 88715, adult male collected by Sebastian Lotzkat and Andreas Hertz on 14 May 2008 at Trail to Rio Hornito, Chiriquí province, Panama (8.67385, -82.21845; 1320 m a.s.l.). SMF 89597, adult male collected by Leonhard Stadler and Nadim Hamad on 6 August 2008 at Cerro Mariposa, Veraguas province, Panama (8.51463, -81.11927; 1003 m a.s.l.). SMF 89786, adult male collected by Sebastian Lotzkat and Andreas Hertz on 1 April 2009 at Cerro Negro, Veraguas province, Panama (8.56901, -81.09894; 900 m a.s.l.). SMF 90024, adult male collected by Arcadio Carrizo on 29 July 2008 at Cerro Negro, Veraguas province, Panama (8.57697, -81.09705; 1085 m a.s.l.). SMF 90207, adult male collected by Sebastian Lotzkat and Andreas Hertz on 29 October 2009 at Cabeceras del Río Chiriquí Mali, Comarca Ngäbe-Buglé, Panama (8.78906, -82.21547; 1054 m a.s.l.). SMF 91579, adult male collected by Sebastian Lotzkat and Andreas Hertz on 11 June 2010 at Cabeceras del Río Chiriquí Mali, Comarca Ngäbe-Buglé, Panama (8.78906, -82.21547; 1054 m a.s.l.). SMF 91580, adult male collected by Sebastian Lotzkat and Andreas Hertz on 24 June 2010 at Bosque Guayabito, Comarca Ngäbe-Buglé, Panama (8.54939, -81.48467; 1510 m a.s.l.).

#### Diagnosis.

*Siboncanopy* sp. nov. is placed in the genus *Sibon* based on phylogenetic evidence (Fig. 1a) and on having the penultimate supralabial conspicuously higher than all other supralabials. The species is diagnosed based on the following combination of characters: (1) 15/15/15 smooth dorsals with enlarged vertebral row (1.4× as wide as adjacent rows); (2) loreal and prefrontal in contact with orbit; (3) 7–8 supralabials with, usually, 4^th^, 5^th^, and occasionally 6^th^ contacting orbit; (4) usually 7–8 infralabials with 2^nd^–6^th^ in contact with chinshields, first pair of infralabials not in contact behind symphysial due to presence of a postmental; (5) 180–189 ventrals in males, 170–185 in females; (6) 113–130 divided subcaudals in males, 107–124 in females; (7) dorsal background color olive with maroon bands (1–2 dorsal scales long mid-dorsally and 3–5 dorsal scales long on the lower flanks) and a reddish tint along the vertebral line (Fig. 2c), ventral surfaces white with encroachment from the dorsal maroon blotches, dorsal aspect of head composed of broad irregular maroon to blackish blotches interspersed with olive to red blotches (Fig. 5b), throat white with brownish blotches, iris dark reddish brown; (8) 336–427 mm SVL in males, 318–357 mm in females; (9) 160–221 mm TL in males, 157–185 mm in females.

#### Comparisons.

*Siboncanopy* sp. nov. is compared to other species of *Sibon* previously subsumed under *S.annulatus* sensu lato (differences summarized in Table 2). From *S.annulatus* sensu stricto, the new species differs in having a single postmental scale, olive spaces among dorsal bands enclosing maroon blotches (Figs 2c, 4a), and by having small irregular (rather than broad and symmetrical) markings on the dorsal surface of the head (Fig. 5). *Siboncanopy* sp. nov. differs from *S.irmelindicaprioae* sp. nov. by having one postmental scale (instead of two), a lower number of infralabials (6–10 vs. 8–10), a lower number of ventrals in males (180–189 vs. 187–196), a different pattern on the dorsal surface of the head (Fig. 5), and by having maroon spots enclosed in the dorsal olive interspaces (Figs 2, 4). *Siboncanopy* sp. nov. differs from *S.marleyae* sp. nov. by having one postmental scale (instead of two), olive spaces among dorsal bands enclosing maroon blotches (Figs 2, 4), and by having irregular (rather than symmetrical) markings on the dorsal surface of the head (Fig. 5).

#### Description of holotype.

Adult female, SVL 321 mm, tail length 157 mm (48% SVL); head length 15.4 mm (4.7% SVL) from tip of snout to commissure of mouth; head width 8.0 mm (76% head length) taken at broadest point; snout-orbit distance 3.3 mm; head distinct from neck; snout short, blunt in dorsal outline and rounded in profile; rostral 2.1 mm wide, higher than broad; internasals 1.6 mm wide, broader than long; prefrontals 1.9 mm wide, longer than broad, entering orbit; supraocular 3.7 mm long, longer than broad; frontal 3.2 mm long, pentagonal and with a straight anterior border, in contact with prefrontals, supraoculars, and parietals; parietals 5.2 mm long, longer than broad; nasal divided, in contact with first three supralabials, loreal, prefrontal, internasal, and rostral; loreal 1.7 mm long, longer than high, entering the orbit; eye diameter 3.0 mm; pupil semi-elliptical; no preocular; two postoculars; temporals 1+2; eight supralabials with 5^th^ and 6^th^ contacting orbit on the right side, seven supralabials with 4^th^ and 5^th^ contacting orbit on the left side; symphysial in contact with chinshields; nine infralabials with 2^nd^–5^th^ contacting chinshields; two pair of chinshields longer than wide; dorsal scales in 15/15/15 rows, smooth, without apical pits; 172 ventrals; 93+ divided subcaudals; cloacal plate entire.

#### Natural history.

Lotzkat (2014) found specimens of *Siboncanopy* sp. nov. foraging at night on vegetation 50–300 cm above the ground in old-growth to moderately disturbed evergreen foothill/montane forests. At Cerro Gaital, Coclé province, we found two specimens moving on mossy branches and moist leaves 40–220 cm above the ground in primary forest during a drizzle. Ray et al. (2012) found this species to be more common in forest and along streams rather than around ponds. Only one individual (a juvenile) was seen crawling along a stream bed. Ray et al. (2012) found oligochaete and mollusk remains in fecal samples of 37 individuals of *S.canopy* sp. nov. from El Copé and Altos del María, Panama. They also observed an individual feeding on a snail at El Copé.

#### Distribution.

*Siboncanopy* sp. nov. is known from 25 localities (listed in Suppl. material 2) in both the Atlantic and Pacific slopes of the Cordillera Central in western Panama, with a population on the slopes of El Valle Volcano. The species occurs over an estimated area of 8,089 km^2^ and has been recorded at elevations 543–1641 m above sea level (Fig. 6).

#### Etymology.

The specific epithet *canopy* is used as a noun in apposition and honors the Canopy Family system of reserves, particularly its Canopy Lodge in Valle de Antón, Coclé province, Panama, where the new species occurs. Though best known for its world-class eco-tourism focused on birds, the Canopy Family also protects habitat that is critical for dozens of poorly studied Panamanian snakes such as *S.canopy* sp. nov. and *S.irmelindicaprioae* sp. nov. The project was founded in 1994 by Raúl Arias de Para and Denise Barakat de Arias, two champions of Panamanian conservation who are deeply intertwined with the Political history of the country. In 2019, the Canopy Family invited us to explore their system of reserves in order to discover their herpetofauna. As a result of this invitation, both *S.canopy* sp. nov. and a new species of *Dipsas* were discovered.

#### Conservation status.

We consider *Siboncanopy* sp. nov. to be included in the Near Threatened category following IUCN Red List criteria (IUCN 2001) because, although the species’ estimated extent of occurrence is less than 10,000 km^2^ and nearly 40% of this area has already been deforested (Fig. 6; CATHALAC 2011), the species occurs in at least four major national parks (Lotzkat 2014) and satellite images show that there is forest connectivity between populations. At Parque Nacional G. D. Omar Torríjos Herrera, the occurrence rates of *S.canopy* sp. nov. have actually increased by a factor of three in the period between 2006 and 2012 (Zipkin et al. 2020). However, the body condition of the individuals in this locality declined following the collapse of amphibian populations due to chytridiomycosis (Zipkin et al. 2020). The status and trend of other populations should be evaluated carefully given that *S.canopy* sp. nov. is endemic to Panama (but see Discussion) and probably highly dependent on old-growth forests.

### ﻿ Sibon marleyae sp. nov.

Taxon classificationAnimaliaSquamataColubridae

3661D1DA-68CF-50E5-B6B6-88B7AF5FB315

https://zoobank.org/86EE4400-A3F1-4414-AB46-5E391D2AED24

**Figure 8.**
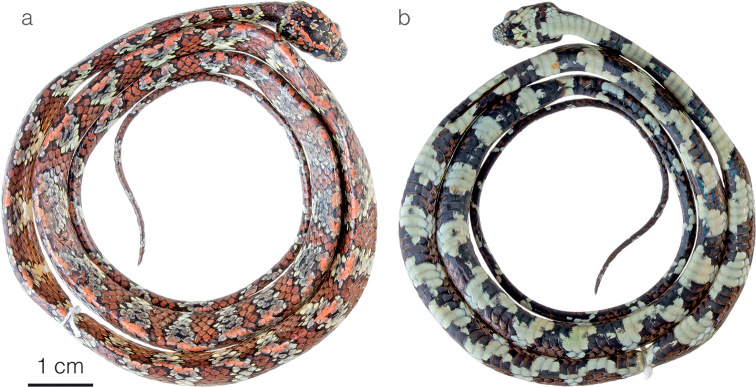
Adult male holotype of *Sibonmarleyae* sp. nov. ZSFQ 5065 in **a** dorsal and **b** ventral view.

Figs 2g–i , 4c , 5d , 8 Proposed standard English name: Marley’s Snail-eating Snake Proposed standard Spanish name: Culebra caracolera de Marley

#### Type material.

***Holotype***: ZSFQ 5065 (Figs 2g, 5d, 8), adult male collected by Amanda Quezada, Eric Osterman, and Regdy Vera in December 2021 at Verdecanandé, Esmeraldas Province, Ecuador (0.52395, -79.01233; 344 m a.s.l.).

***Paratypes***: MZUTI 3034, adult male collected by Jaime Culebras on 22 July 2013 at Reserva Itapoa, Esmeraldas Province, Ecuador (0.51307, -79.13401; 321 m a.s.l.). ICN 10834, adult male collected at San José del Palmar, Chocó department, Colombia (4.96841, -76.22751; 1338 m a.s.l.). ZSFQ 5069, adult male collected by Jose Vieira, Daniela Franco, and Alex Mora on 4 December 2019 at Reserva Biológica Canandé, Esmeraldas Province, Ecuador (0.49531, -79.17832; 560 m a.s.l.). ZSFQ 5067, adult female collected by Jose Vieira, Frank Pichardo, and Matteo Espinosa at Durango, Esmeraldas Province, Ecuador (1.04161, -78.62658; 245 m a.s.l.). CPZ-UV 04567, adult female collected by Andrés Gómez Figueroa at Tamboral, Valle del Cauca department, Colombia. CZI-R009, adult female collected by Santiago Orozco on 9 September 2018 at Campamento Yatacué, Valle del Cauca department, Colombia (3.57472, -76.87777; 598 m a.s.l.). CZI-R051, adult female collected by Santiago Orozco on 16 June 2019 at Represa Murrapal, Valle del Cauca department, Colombia (3.55283, -76.98077; 321 m a.s.l.).

#### Diagnosis.

*Sibonmarleyae* sp. nov. is placed in the genus *Sibon* based on phylogenetic evidence (Fig. 1a) and on having the penultimate supralabial conspicuously higher than all other supralabials. The species is diagnosed based on the following combination of characters: (1) 15/15/15 smooth dorsals with enlarged vertebral row (up to 2× as wide as adjacent rows); (2) loreal and prefrontal in contact with orbit; (3) 7–8 supralabials with, usually, 4^th^, 5^th^, and occasionally 6^th^ contacting orbit; (4) usually 8–9 infralabials with 2^nd^–6^th^ in contact with chinshields, first pair of infralabials not in contact behind symphysial due to presence of two postmentals; (5) 186–204 ventrals in males, 176–193 in females; (6) 130–143 divided subcaudals in males, 109–128 in females; (7) dorsal background color olive to yellow with maroon (black in juveniles) bands (1–2 dorsal scales long mid-dorsally and 3–5 dorsal scales long on the lower flanks) and a reddish tint along the vertebral line (Figs 2g–i, 4c), ventral surfaces white with encroachment from the dorsal maroon blotches (Fig. 8b), dorsal aspect of head composed of blackish symmetrical markings on a red background color (Fig. 5d), throat white with broad brownish blotches, iris rich reddish brown; (8) 308–464 mm SVL in males, 329–368 mm in females; (9) 167–233 mm TL in males, 175–183 mm in females.

#### Comparisons.

*Sibonmarleyae* sp. nov. is compared to other species of *Sibon* previously subsumed under *S.annulatus* sensu lato (differences summarized in Table 2). From *S.annulatus* sensu stricto, the new species differs in having maroon bands usually broken along vertebral line rather than bands extending over the entire dorsal and lateral surfaces, a bright reddish coloration along the mid-dorsum and on the top of the head (Figs 4c, 5d, 8a), and a higher number of ventral scales in males and females (Table 2). *Sibonmarleyae* sp. nov. differs from *S.canopy* sp. nov. by having two postmental scales (instead of only one), a higher number of ventrals in males and females, a pattern of symmetrical (rather than irregular and asymmetrical) markings on the dorsal surface of the head (Fig. 5), and by lacking maroon spots enclosed in the dorsal olive interspaces (Figs 2g–i, 4c). *Sibonmarleyae* sp. nov. differs from *S.irmelindicaprioae* sp. nov. primarily by having a pattern of broad blackish markings on the head instead of a finely variegated pattern and by having a higher number of ventrals and subcaudals in both males and females (Table 2).

#### Description of holotype.

Adult male, SVL 335 mm, tail length 167 mm (49.8% SVL); head length 12.8 mm (3.8% SVL) from tip of snout to angle of jaw; head width 7.6 mm (59% head length) taken at broadest point; snout-orbit distance 3.1 mm; head distinct from neck; snout short, blunt in dorsal outline and rounded in profile; rostral 2.1 mm wide, higher than broad; internasals 1.3 mm wide, broader than long; prefrontals 1.6 mm wide, longer than broad, entering orbit; supraocular 2.8 mm long, longer than broad; frontal 2.9 mm long, pentagonal and with a straight anterior border, in contact with prefrontals, supraoculars, and parietals; parietals 4.3 mm long, longer than broad; nasal divided, in contact with two supralabials, loreal, prefrontal, internasal, and rostral; loreal 1.2 mm long, longer than high, entering the orbit; eye diameter 2.9 mm; pupil semi-elliptical; no preocular; two postoculars; temporals 1+2; seven supralabials with 4^th^ and 5^th^ contacting orbit; symphysial precluded from contacting chinshields by the presence of two small postmentals; eight infralabials with 2^nd^–6^th^ contacting chinshields on the right side, nine infralabials with 2^nd^–7^th^ contacting chinshields on the left side; two pairs of chinshields longer than wide; dorsal scales in 15/15/15 rows, smooth, without apical pits; 204 ventrals; 132 divided subcaudals; cloacal plate entire.

#### Natural history.

Specimens of *Sibonmarleyae* sp. nov. have been found at night foraging on shrubs and trees 1–6 m above the ground in old-growth evergreen lowland/foothill forests, particularly along streams and small rivers. Snakes of this species are docile and never attempt to bite. When threatened, individuals may hide the head among body coils and produce a musky and distasteful odor. One female (Fig. 2h) from the type locality laid two eggs in a terrarium. After an incubation period of 80 days, one of the eggs hatched (Fig. 2i).

#### Distribution.

*Sibonmarleyae* sp. nov. is known from 17 localities (listed in Suppl. material 2) along the Chocoan lowlands and adjacent foothills of the Andes in Ecuador and Colombia, with populations on the coastal mountain ranges Mache-Chindul and Cerro Pata de Pájaro in Ecuador. The species has been recorded at elevations 131–1338 m above sea level (Fig. 6).

#### Etymology.

The specific epithet *marleyae* is a patronym honoring a young nature lover, Marley Sheth, the 11-year old daughter of Brian and Adria Sheth, both long-time supporters of biodiversity conservation around the world.

#### Conservation status.

We consider *Sibonmarleyae* sp. nov. to be included in the Least Concern category following IUCN Red List criteria (IUCN 2001) because the species is distributed over a region of the Chocó biome that holds large areas of continuous unspoiled forest. Based on the species distribution model presented in Fig. 6 in combination with maps of vegetation cover of Colombia (IDEAM 2014) and Ecuador (MAE 2012), we estimate that more than half (~ 55%) of the species’ forest habitat is still standing. Unfortunately, vast areas of the Chocó rainforest in western Ecuador have already been converted to pastures (Myers et al. 2000). However, *S.marleyae* sp. nov. occurs over an area greater than 25,000 km^2^ and is presumably not declining fast enough to qualify for a threatened category.

### ﻿ Sibon vieirai sp. nov.

Taxon classificationAnimaliaSquamataColubridae

5413F02B-73CA-5BCD-B81B-A3D733EDEC50

https://zoobank.org/AEE40456-E5FB-492E-822E-C8667E6874B6

**Figure 9.**
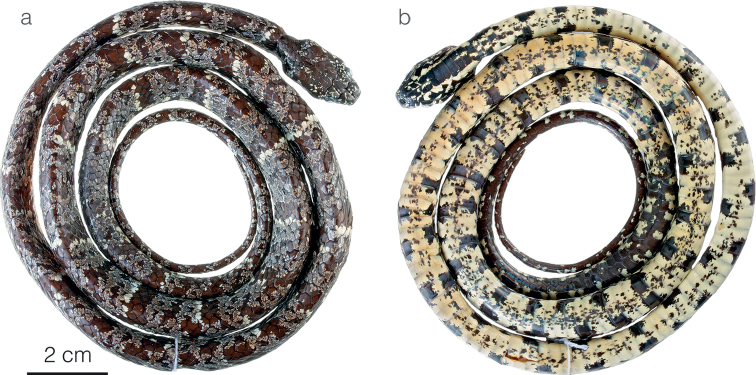
Adult male holotype of *Sibonvieirai* sp. nov. ZSFQ 5071 in **a** dorsal and **b** ventral view.

**Figure 10.**
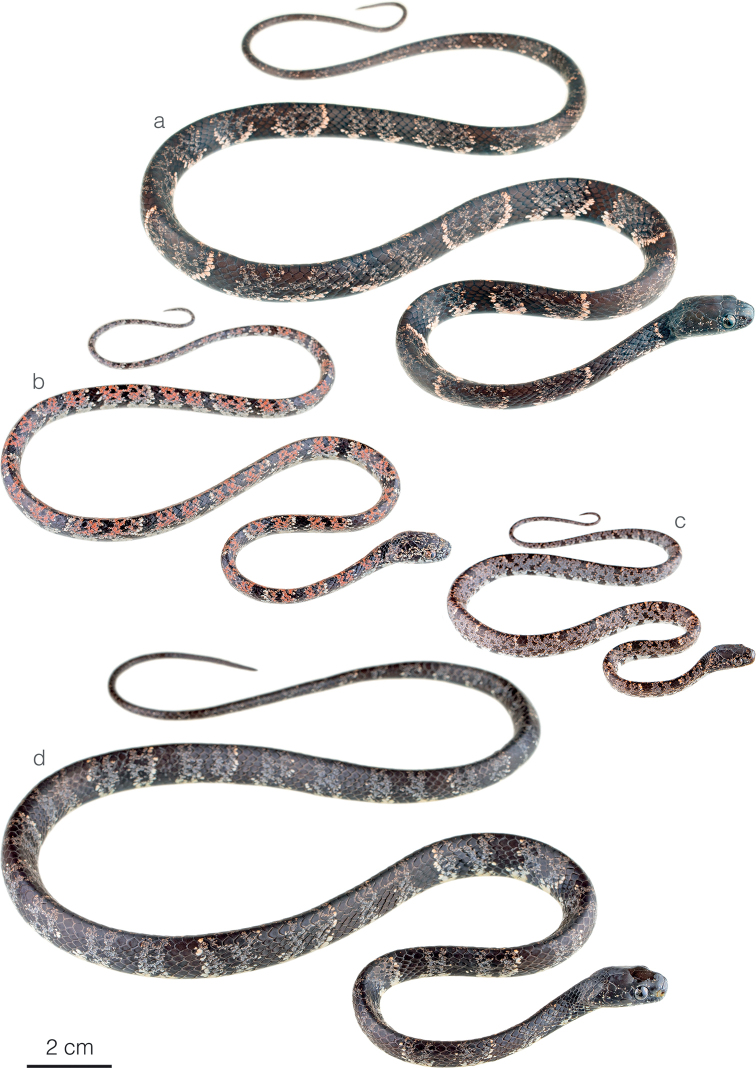
Photographs of species of the *Sibonnebulatusleucomelas* complex in life **a***S.leucomelas* from Morromico Reserve, Chocó department, Colombia **b***S.vieirai* sp. nov. ZSFQ 5073 from Hacienda Cerro Chico, Los Ríos province, Ecuador **c***S.vieirai* sp. nov. from Tundaloma Lodge, Esmeraldas Province, Ecuador **d***S.vieirai* sp. nov. holotype ZSFQ 5071 from Tundaloma Lodge, Esmeraldas Province, Ecuador.

**Figure 11.**
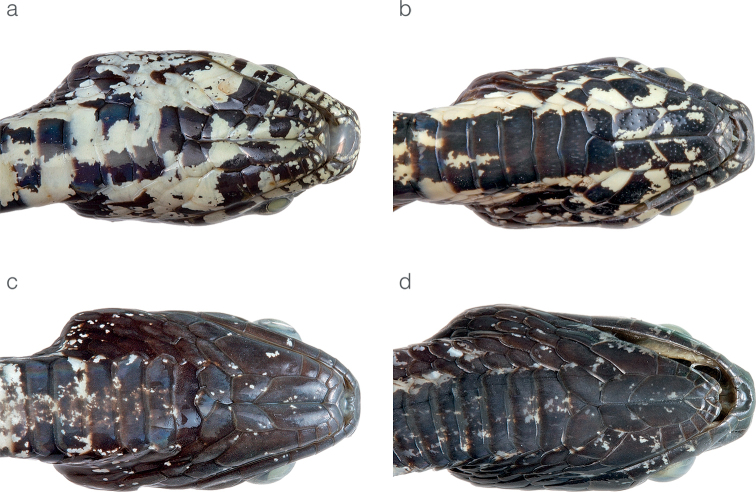
Differences in throat color pattern between species of the *Sibonnebulatusleucomelas* complex **a***S.vieirai* sp. nov. ZSFQ 5073 from Hacienda Cerro Chico, Los Ríos province, Ecuador **b***S.vieirai* sp. nov. holotype ZSFQ 5071 from Tundaloma Lodge, Esmeraldas Province, Ecuador **c***S.leucomelas*CZI-R075 from Represa Murrapal, Valle del Cauca department, Colombia **d***S.leucomelas*CZI-R029 from Campamento Yatacué, Valle del Cauca department, Colombia.

Figs 4d , 9 , 10b–d , 11a, b Proposed standard English name: Vieira’s Snail-eating Snake Proposed standard Spanish name: Culebra caracolera de Jose Vieira

#### Type material.

***Holotype***: ZSFQ 5071 (Figs 9, 10d, 11b), adult male collected by Jose Vieira, Frank Pichardo, and Matteo Espinosa on 28 February 2021 at Tundaloma Lodge, Esmeraldas Province, Ecuador (1.18166, -78.74945; 74 m a.s.l.).

***Paratypes***: MZUA.RE.0328, adult male collected at Jauneche, Los Ríos province, Ecuador (-1.33333, -79.58333; 41 m a.s.l.). MZUTI 4810, adult female collected by Jaime Culebras on 14 February 2016 at Bosque Privado El Jardín de los Sueños, Cotopaxi province, Ecuador (-0.83142, -79.21337; 349 m a.s.l.). ZSFQ 5070, adult male collected by Alejandro Arteaga and Jose Vieira on 12 March 2018 at La Primavera, Carchi province, Ecuador (0.79502, -78.21763; 1228 m a.s.l.). MZUTI 3911, juvenile male collected by Jaime Culebras on 11 November 2014 at Itapoa Reserve, Esmeraldas Province, Ecuador (0.51307, -79.13401; 321 m a.s.l.). MZUTI 5342, adult male collected by Jorge Vaca on 27 May 2017 at Reserva Jama Coaque, Manabí province, Ecuador (-0.11556, -80.12472; 299 m a.s.l.). ZSFQ 5073, adult male collected by Jose Vieira on 23 August 2020 at Hacienda Cerro Chico, Los Ríos province, Ecuador (-0.63862, -79.42585; 141 m a.s.l.). USNM 285499, juvenile male collected by Roy McDiarmid on 1 January 1979 at Centro Científico Río Palenque, Los Ríos province, Ecuador (-0.58333, -79.36667; 173 m a.s.l.). USNM 285501, adult male collected by Roy McDiarmid on 10 March 1979 at Hacienda Cerro Chico, Los Ríos province, Ecuador (-0.63862, -79.42585; 141 m a.s.l.). USNM 283534, adult of undetermined sex collected on 6 June 1981 at Rancho Santa Teresita, Santo Domingo de los Tsáchilas province, Ecuador (-0.25277, -79.37946; 288 m a.s.l.). USNM 285498, adult of undetermined sex collected by Roy McDiarmid on 23 May 1976 at Centro Científico Río Palenque, Los Ríos province, Ecuador (-0.58333, -79.36667; 173 m a.s.l.). USNM 285500, adult of undetermined sex collected by Roy McDiarmid on 2 February 1976 at Centro Científico Río Palenque, Los Ríos province, Ecuador (-0.58333, -79.36667; 173 m a.s.l.). MZUA.RE.0174, adult female collected at Macul, Los Ríos province, Ecuador (-1.1298, -79.65731; 65 m a.s.l.).

#### Diagnosis.

*Sibonvieirai* sp. nov. is placed in the genus *Sibon* based on phylogenetic evidence (Fig. 1a) and on having the penultimate supralabial conspicuously higher than all other supralabials. The species is diagnosed based on the following combination of characters: (1) 15/15/15 smooth dorsals with enlarged vertebral row (1.4–3× as wide as adjacent rows); (2) loreal and prefrontal in contact with orbit; (3) 7–8 supralabials with, usually, 4^th^, 5^th^, and occasionally 6^th^ contacting orbit; (4) 9–10 infralabials with 1^st^ to 6^th^ in contact with chinshields, first pair of infralabials in contact behind symphysial; (5) 183–195 ventrals in males, 178–189 in females; (6) 95–105 divided subcaudals in males, 78–92 in females; (7) dorsal background color slate gray to brownish gray with faint blackish blotches or incomplete bands bordered by a series of white dots, fine blackish and white speckling in the interspaces (Figs 4d, 9a, 10b–d), ventral surfaces white with encroachment from the dorsal blackish blotches and with or without fine black speckles in-between the blotches (Fig. 9b), dorsal aspect of head black with fine white speckles, throat white with various amounts of black markings that form a checkerboard pattern (Fig. 11a, b), iris pale gray finely variegated with black; (8) 363–542 mm SVL in males, 352–544 mm in females; (9) 127–224 mm TL in males, 113–170 mm in females.

#### Comparisons.

*Sibonvieirai* sp. nov. is most similar to *S.leucomelas*, from which it differs primarily on the basis of coloration (differences summarized under Table 3). In *S.vieirai* sp. nov. (Figs 4d, 10b–d), the complete black and pale dorsal bands typical of *S.leucomelas* (Fig. 10a) are usually absent. Instead, the white “bands” are formed by series of white spots and the black bands are faint and incomplete. The color of the pale “bands” also differs between species: rosy white in S. *leucomelas* and white in *S.vieirai* sp. nov. (Fig. 10). In *S.vieirai* sp. nov the throat has a checkerboard pattern of black and white markings whereas in *S.leucomelas* it is entirely black with fine white speckling (Fig. 11). Overall, specimens assignable to *S.leucomelas* have a greater number of ventral scales than *Sibonvieirai* sp. nov. in both males and females, although there is overlap in the counts (Table 3). *Sibonvieirai* sp. nov. differs from *S.bevridgelyi* by having white (instead of golden yellow) dorsal markings on a primarily gray (instead of rusty brown to deep maroon) background color. Arteaga at al. (2018) presented an in-depth comparison between *S.bevridgelyi* and *Sibonvieirai* sp. nov. (reported as *S.nebulatus* from Ecuador).

**Table 3.** Differences in coloration, scale counts, and size between *Sibonleucomelas* and *S.vieirai* sp. nov. The range of each continuous variable is from our own sample, Boulenger 1896, and Frazier et al. (2006). The numbers in parentheses represent the sample size.

Variable	*Sibonleucomelas*		*Sibonvieirai* sp. nov.	
White dorsal bands	Distinct, 1–2 scales wide		Incomplete, broken into dots	
Complete black bands extending over the entire dorsal and lateral surfaces	Present, distinct		Usually absent; if present, indistinct and broken	
Color of pale dorsal markings	Rosy white		White	
Throat pattern	Entirely black with fine white speckling		Checkerboard, with large black and white markings	
Sex	Males (*n* = 5)	Females (*n* = 7)	Males (*n* = 8)	Females (*n* = 5)
Maximum TOL	809 mm	700 mm	732 mm	714 mm
Ventral scales	190–198	187–194	183–195	178–189
Subcaudal scales	86–101	84–100	95–105	78–92

#### Description of holotype.

Adult male, SVL 515 mm, tail length 199 mm (38.6% SVL); head length 20.7 mm (4.0% SVL) from tip of snout to angle of jaw; head width 11.6 mm (55% head length) taken at broadest point; snout-orbit distance 4.9 mm; head distinct from neck; snout short, blunt in dorsal outline and rounded in profile; rostral 3.8 mm wide, higher than broad; internasals 2.1 mm wide, broader than long; prefrontals 3.4 mm wide, slightly broader than long, entering orbit; supraocular 3.6 mm long, longer than broad; frontal 4.3 mm long, with a rounded triangular shape, in contact with prefrontals, supraoculars, and parietals; parietals 6.4 mm long, longer than broad; nasal divided, in contact with two supralabials, loreal, prefrontal, internasal, and rostral; loreal 2.3 mm long, longer than high, entering the orbit; eye diameter 3.9 mm; pupil semi-elliptical; no preocular; two postoculars; temporals 1+2; seven supralabials with 4^th^ and 5^th^ contacting orbit; symphysial precluded from contacting chinshields by first pair of infralabials; nine infralabials with 1^st^–6^th^ contacting chinshields; two pairs of chinshields longer than wide; dorsal scales in 15/15/15 rows, smooth, without apical pits; 195 ventrals; 105 divided subcaudals; cloacal plate entire.

#### Natural history.

Specimens of *Sibonvieirai* sp. nov. have been found in old growth to heavily disturbed evergreen lowland/foothill forests as well as in rural gardens and plantations. Active snakes have been seen at night foraging at ground level or on vegetation up to 3 m above the ground. One snake was spotted as it emerged from under a pile of logs at sunset. Based on our own field experience, individuals appear to be more active when it is raining or drizzling. In the field in Ecuador, specimens of *S.vieirai* sp. nov. have been observed feeding on slugs and snails. A female from Hostería Selva Virgen, Pichincha province, Ecuador laid a clutch of four eggs.

#### Distribution.

**Figure 12.**
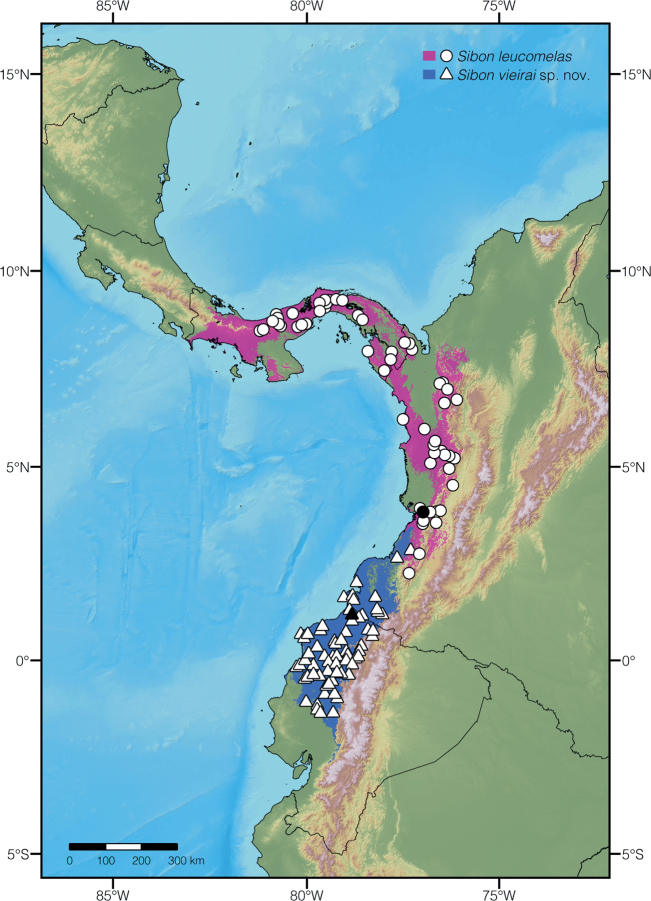
Distribution of species of *Sibon* previously subsumed under *S.nebulatusleucomelas*. Black symbols represent type localities; white symbols other localities listed in Suppl. material 2. Colored areas show the extent of suitable environmental conditions for each species.

*Sibonvieirai* sp. nov. is known from at least 95 localities (listed in Suppl. material 2) along the Chocoan lowlands and adjacent foothills of the Andes in northwestern Ecuador and southwestern Colombia. Previous records of *S.nebulatus* from the rainforests of northwestern Ecuador as well as those of the Pacific lowlands of Colombia in Nariño department almost surely correspond to this new species. *Sibonvieirai* sp. nov. occurs over an estimated 58,551 km^2^ area and has been recorded at elevations 5–1803 m above sea level (Fig. 12).

#### Etymology.

The specific epithet *vieirai* is a patronym honoring Jose Vieira, a Venezuelan biologist and wildlife photographer who created the Ex-Situ project, a free-access photo bank depicting Latin American fauna on a white background. Jose Vieira’s photos have been crucial in illustrating field guides about herpetofauna, educational posters, and research publications. Most of the images in this work were created by Jose Vieira. Additionally, after nearly six years of active collaboration with one of us (AA), it has become evident that Jose is one of the most tireless and focused young field biologists ever to sample the jungles of the tropics, a work ethic that has resulted in the generation of photo and museum vouchers for hundreds of poorly studied species of herpetofauna, including the holotype of this new *Sibon*.

#### Conservation status.

We consider *Sibonvieirai* sp. nov. to be included in the Least Concern category following IUCN Red List criteria (IUCN 2001) because the species is distributed over a region of the Chocó biome that holds large areas of continuous unspoiled forest. Based on the species distribution model presented in Fig. 12 in combination with maps of vegetation cover of Colombia (IDEAM 2014) and Ecuador (MAE 2012), we estimate that more than half (~ 51%) of the species’ forest habitat is still standing. Unfortunately, vast areas of the Chocó rainforest in western Ecuador have already been converted to pastures (Myers et al. 2000). However, *S.vieirai* sp. nov. occurs over an area greater than 50,000 km^2^ and is presumably not declining fast enough to qualify for a threatened category.

### ﻿ Dipsas welborni sp. nov.

Taxon classificationAnimaliaSquamataColubridae

452868F0-F4B8-572A-8F34-A792D5F30810

https://zoobank.org/8F1C3963-FB25-4C98-81E9-714727A4CCEF

**Figure 13.**
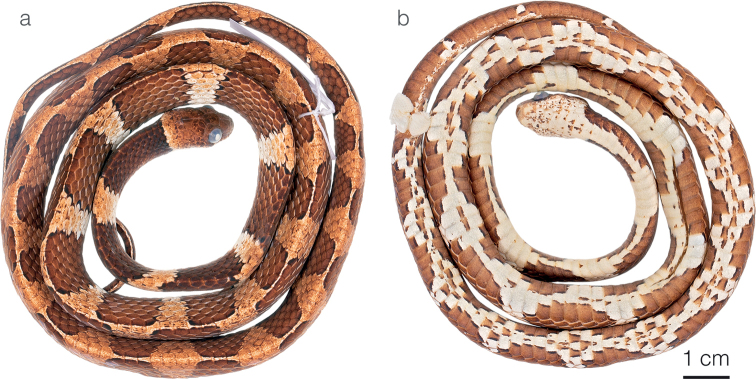
Male holotype of *Dipsaswelborni* sp. nov. MZUTI 3663 in **a** dorsal and **b** ventral view.

**Figure 14.**
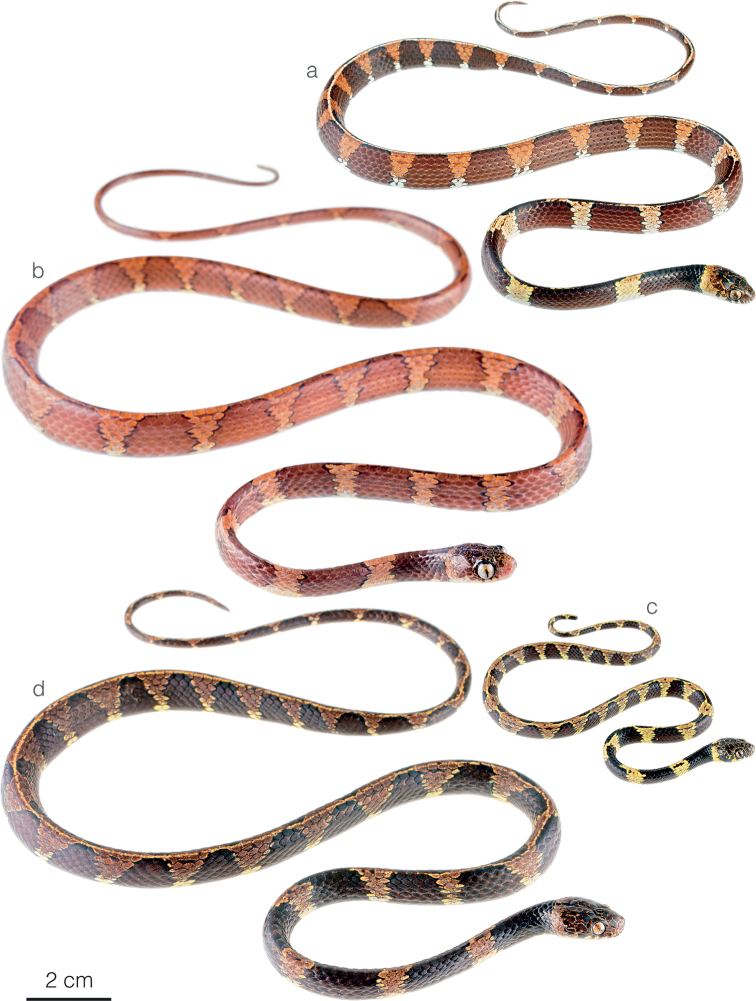
Photographs of species of *Dipsas* previously subsumed under *D.vermiculata***a***D.welborni* sp. nov. paratype ZSFQ 5060 from Vía a Nuevo Paraíso, Zamora Chinchipe province, Ecuador **b***D.vermiculata*ZSFQ 5059 from Tamandúa Reserve, Pastaza province, Ecuador **c***D.vermiculata*ZSFQ 5064 from Narupa Reserve, Napo province, Ecuador **d***D.vermiculata*ZSFQ 5061 from Narupa Reserve, Napo province, Ecuador.

**Figure 15.**
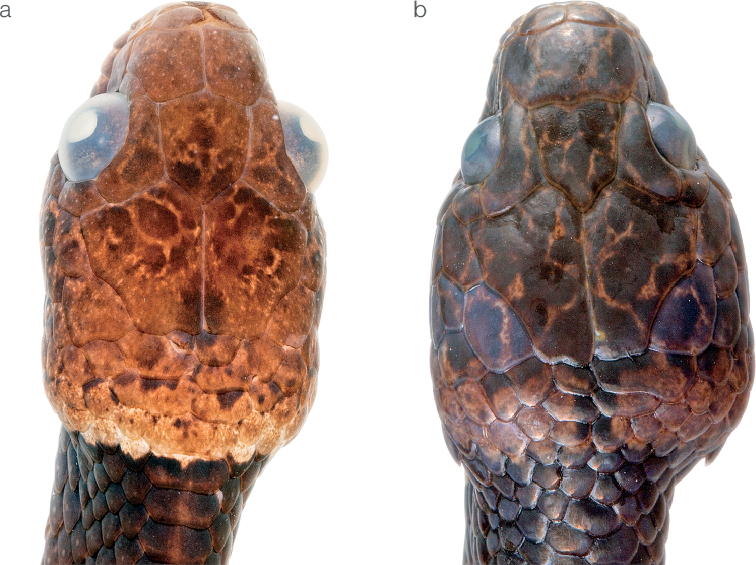
Difference in the condition of the prefrontal scale between *Dipsaswelborni* sp. nov. and *D.vermiculata***a** divided in *Dipsaswelborni* sp. nov. holotype MZUTI 3663 **b** fused in *D.vermiculata*ZSFQ 5061.

Figs 4f , 13 , 14a , 15a Proposed standard English name: Welborn’s Snail-eating Snake Proposed standard Spanish name: Culebra caracolera de Welborn

#### Type material.

***Holotype***: MZUTI 3663 (Figs 13, 15a), adult male collected on 2 July 2014 at Reserva Maycu, Zamora Chinchipe province, Ecuador (-4.20719, -78.63987; 960 m a.s.l.).

***Paratypes***: MZUA.RE.0261, adult male collected at Nangaritza, Zamora Chinchipe province, Ecuador (-4.43169, -78.63869; 996 m a.s.l.). DHMECN 11197, juvenile male collected by Raquel Betancourt and Miguel Alcoser on 18 September 2012 at Concesión ECSA, Zamora Chinchipe province, Ecuador (-3.57245, -78.46982; 790 m a.s.l.). ZSFQ 5060 (Figs 4f, 14a), female collected by Alejandro Arteaga and Amanda Quezada at Maycu Reserve, Zamora Chinchipe province, Ecuador (-4.26395, -78.64483; 1078 m a.s.l.).

#### Diagnosis.

*Dipsaswelborni* sp. nov. is placed in the genus *Dipsas* based on phylogenetic evidence (Fig. 1b) and the absence of a labial that is noticeably higher than other labials. The species is diagnosed based on the following combination of characters: (1) 13/13/13 smooth dorsals with enlarged vertebral row (1.8–2.1× as wide as adjacent rows); (2) loreal and a preocular in contact with orbit; (3) 7–8 supralabials with 4^th^, 5^th^, and occasionally 6^th^, contacting orbit; (4) 8–9 infralabials with 1^st^ to 5^th^ or to 6^th^ in contact with chinshields, one pair of infralabials in contact behind symphysial; (5) 181–193 ventrals in males, 177–179 in females; (6) 107–116 divided subcaudals in males, 105–106 in females; (7) dorsal color consisting of 21–26 dark brown to blackish body blotches (8–13 dorsal scales long anteriorly and 2–5 dorsal scales long posteriorly) separated from each other by narrow (2–3 dorsal scales long) pale brown interspaces that become white towards the lower lateral side, ventral surfaces white with encroachment from the dorsal dark brown blotches and with smaller brownish marks in-between the blotches, dorsal aspect of head dark reddish brown with fine bright yellow (juveniles) to light brown (adults; Fig. 15a) reticulations, throat white, iris pale brown; (8) 491–542 mm SVL in males, 321–595 mm in females; (9) 190–279 mm TL in males, 132–281 mm in females.

#### Comparisons.

*Dipsaswelborni* sp. nov. differs from the majority of its congeners by having dorsal scales arranged in 13/13/13 rows, loreal entering the orbit, and dorsum of head strongly vermiculated. The new species is most similar to *D.vermiculata*, from which it differs on the basis of the following characters of coloration and lepidosis (Fig. 14; Table 4). In *D.welborni* sp. nov., there are two prefrontal scales (partially fused in ZSFQ 5060) whereas in all specimens of *D.vermiculata* examined (Suppl. material 1) as well as the four Ecuadorian specimens reported in Peters (1960), the prefrontals are fused into a single scale (Fig. 15). Females of *Dipsaswelborni* sp. nov. have more ventrals (177–179) and subcaudals (105–106) than those of *D.vermiculata* (173–174 ventrals and 99–103 subcaudals). Females of the new species attain a larger body size than those of *D.vermiculata* (Table 4), and males of the former also have more rows of spines on the asulcate surface of the hemipenis body (the hemipenis of *D.vermiculata* is depicted in Vera-Pérez 2020 whereas the organs of four males of *D.welborni* sp. nov. are depicted in Pazmiño-Otamendi et al. 2020). Finally, the two species further differ in the background color of the ventral surfaces: always white in *D.welborni* sp. nov. (Fig. 13b), and usually yellow or occasionally pale yellowish white in *D.vermiculata*.

**Table 4.** Differences in coloration, scale counts, and size between *Dipsasvermiculata* and *D.welborni* sp. nov. The range of each continuous variable is from our own sample, Peters (1960), and Vera-Pérez (2020). The numbers in parentheses represent the sample size.

Variable	*Dipsasvermiculata*		*Dipsaswelborni* sp. nov.	
Background color of ventral surfaces	Always white		Usually yellow, occasionally pale yellowish white	
Prefrontals fused	No (partially fused in ZSFQ 5060)		Yes	
Rows of spines on hemipenis body (asulcate surface)	1–2 rows of curved spines		2 rows of straight spines followed by 2–3 rows of curved spines	
Sex	Males (*n* = 8)	Females (*n* = 3)	Males (*n* = 7)	Females (*n* = 3)
Maximum SVL	515 mm	501 mm	542 mm	595 mm
Maximum TOL	735 mm	701 mm	689 mm	876 mm
Ventral scales	181–192	173–174	181–193	177–179
Subcaudal scales	103–113	99–103	107–116	105–106

#### Description of holotype.

Adult male, SVL 542 mm, tail length 195 mm (incomplete); head length 16.4 mm (3.0% SVL) from tip of snout to angle of jaw; head width 10.2 mm (62% head length) taken at broadest point; snout-orbit distance 3.8 mm; head distinct from neck; snout short, blunt in dorsal outline and rounded in profile; rostral 2.4 mm wide, higher than broad; internasals 1.8 mm wide, broader than long; prefrontals 2.7 mm wide, longer than broad, not entering orbit; supraocular 3.8 mm long, longer than broad; frontal 4.1 mm long, hexagonal and with angled anterior border, in contact with prefrontals, supraoculars, and parietals; parietals 5.5 mm long, longer than broad; nasal divided, in contact with two supralabials, loreal, prefrontal, internasal, and rostral; loreal 2.1 mm long, longer than high, entering the orbit; eye diameter 3.4 mm; pupil semi-elliptical; one small preocular above loreal; two postoculars; temporals 2+2; seven supralabials with 4^th^–5^th^ contacting orbit; symphysial precluded from contacting chinshields by first pair of infralabials; nine infralabials with 1^st^ to 5^th^ contacting chinshields; three pairs of chinshields, first longer than wide; dorsal scales in 13/13/13 rows, smooth, without apical pits; 185 ventrals; 80+ divided subcaudals; cloacal plate entire.

#### Natural history.

Specimens of *Dipsaswelborni* sp. nov. have been found foraging on vegetation 20–350 cm above the ground in old-growth to moderately disturbed evergreen montane forests. Snakes of this species are docile and never attempt to bite. When threatened, individuals may flatten their body and expand their head to simulate a triangular shape as well as produce a musky and distasteful odor.

#### Distribution.

**Figure 16.**
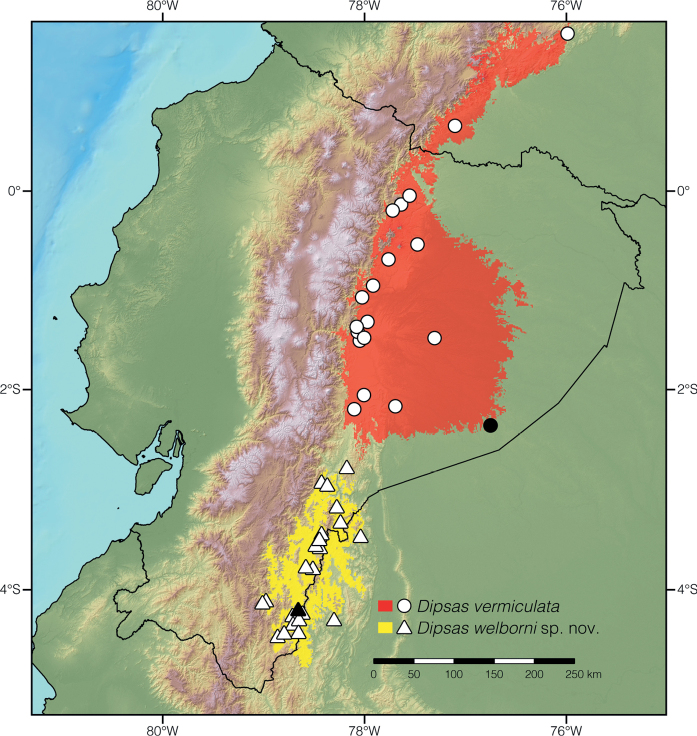
Distribution of species of *Dipsas* previously subsumed under *D.vermiculata*. Black symbols represent type localities; white symbols other localities listed in Suppl. material 2. Colored areas show the extent of suitable environmental conditions for each species.

*Dipsaswelborni* sp. nov. is known from 26 localities (listed in Suppl. material 2) along the Cordillera del Cóndor in southeastern Ecuador (provinces Morona Santiago and Zamora Chinchipe) and northern Peru (Amazonas department). The species occurs over an estimated area of 10,521 km^2^ and has been recorded at elevations 853–1843 m above sea level (Fig. 16). One locality, Etseketai Entse, Amazonas department, Peru, is in the Río Cenepa valley at ~ 245 m above sea level. Since this locality is much lower in elevation than other localities in the Cordillera del Cóndor, it is likely that the specimens collected there (USNM 316599–600) were actually found on the neighboring mountain ridges.

#### Etymology.

The specific epithet *welborni* is a patronym honoring David Welborn, a lifelong champion of ecosystem and species conservation who supports and serves on several nonprofit boards dedicated to the environment. David retired from the board of Nature and Culture International in 2021 after 18 years of service, including four as board chairman. Nature and Culture International, a non-profit organization, has conserved more than 9 million hectares of tropical Latin American ecosystems, including key habitat in the Maycu Reserve of southeastern Ecuador, where *Dipsaswelborni* sp. nov. was discovered.

#### Conservation status.

We consider *Dipsaswelborni* sp. nov. to be in the Near Threatened category following IUCN Red List criteria (IUCN 2001) because the species is distributed over a region of the Amazonian slopes of the Andes that holds large areas of continuous unspoiled forest. Based on the species distribution model presented in Fig. 16 in combination with the most recent maps of vegetation cover of the Amazon basin (MapBiomas Amazonía 2022), we estimate that the majority (~ 76%) of the species’ forest habitat in Ecuador is still standing. Unfortunately, vast areas of the Cordillera del Cóndor, notably on the Ecuadorian part of the species’ range, are being cleared to make room for large-scale opencast mining operations (Chicaiza 2010; Valencia et al. 2017). However, since *D.welborni* sp. nov. occurs over an area greater than 10,000 km^2^, the species does not qualify for a threatened category.

##### ﻿Presence of *Sibonayerbeorum* in Ecuador and Valle del Cauca, Colombia

**Figure 17.**
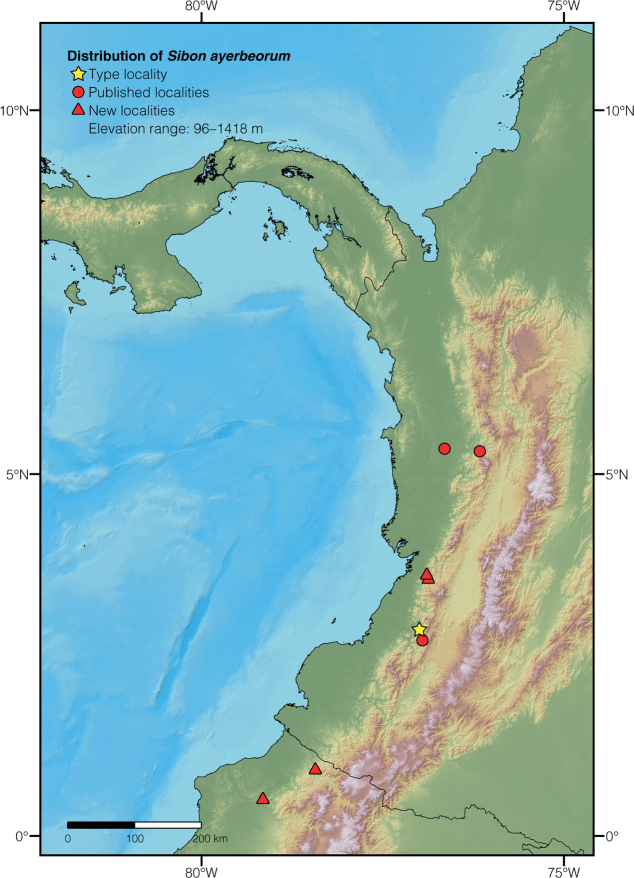
Distribution of *Sibonayerbeorum* showing previously known (circles) and new records (triangles).

We expand the distribution of *Sibonayerbeorum*, a species previously known only from departments Cauca (Vera-Pérez 2019), Chocó (Echavarría-Rentería and Medina-Rangel 2021), and Risaralda (Bonilla and Moya 2021) in Colombia. We examined three additional specimens (listed in Suppl. material 1) at Colección Zoológica de la Universidad ICESI (labeled CZI) and at ZSFQ that represent, respectively, new records for Valle del Cauca department in Colombia and Esmeraldas province in Ecuador. CZI-R063 is a juvenile male collected by Santiago Orozco on 17 August 2019 at La Loca, Valle del Cauca department, Colombia (3.57656, -76.88029; 658 m a.s.l.). CZI-R067 is a juvenile male collected by Santiago Orozco on 30 August 2019 at La Riqueza, Valle del Cauca department, Colombia (3.59874, -76.89184; 621 m a.s.l.). ZSFQ 5066 (Fig. 2f) is a juvenile male collected by Jose Vieira, Daniela Franco, and Alex Mora on 4 December 2019 at Reserva Biológica Canandé, Esmeraldas Province, Ecuador (0.49531, -79.17832; 560 m a.s.l.). These specimens agree in coloration and lepidosis with the description of *S.ayerbeorum* presented in Vera-Pérez 2019 (expanded in Echavarría-Rentería and Medina-Rangel 2021), most notably in having a much lower (fewer than 160) number of ventral scales than any other sympatric *Sibon* species, vertebral scale row not noticeably wider than adjacent rows, postmentals absent, dorsal coloration green to grayish brown with black-bordered reddish markings, and ventral coloration consisting of a checkerboard pattern of yellowish white markings interspersed with blackish markings. We also report an individual of *S.ayerbeorum* photographed (https://www.inaturalist.org/photos/179847493; DHMECN 14936) by Mateo Vega on July 21, 2019 at Comunidad El Baboso, Carchi province, Ecuador (0.89972, -78.44797; 803 m). We did not examine this specimen, but the photograph agrees in coloration with the variation reported for this species in Vera-Pérez (2019) and Echavarría-Rentería and Medina-Rangel (2021). The updated distribution of *S.ayerbeorum* is shown in Fig. 17 and includes both published records as well as new localities reported here (summarized in Suppl. material 2).

## Supplementary Material

XML Treatment for Sibon irmelindicaprioae

XML Treatment for Sibon canopy

XML Treatment for Sibon marleyae

XML Treatment for Sibon vieirai

XML Treatment for Dipsas welborni

## ﻿Appendix 1

**Table A1.** GenBank accession numbers for loci and terminals of taxa and outgroups sampled in this study. Novel sequence data produced in this study are marked with an asterisk (*).

Species	Voucher	12S	16S	COI	CYTB	ND4	DNAH3	NT3
*Atractusukupacha*	QCAZ 4944	–	MH790540	–	MN887689	MN887714	–	–
*Dipsasalbifrons*	MZUSP 13993	JQ598803	JQ598866	–	JQ598925	–	–	–
*Dipsasandiana*	MZUTI 3505	–	MH341010	–	MH374974	–	–	–
*Dipsasarticulata*	USNM 348490	JQ598804	MH140680	MH140122	–	–	–	–
*Dipsasbobridgelyi*	MZUTI 5414	–	MH341016	–	MH374984	–	–	–
*Dipsasbucephala*	GRCOLLI 25659	MH341087	MH341018	–	MH375026	MH375052	–	–
*Dipsascatesbyi*	QCAZ 13558	MH341088	MH341019	–	MH374975	MH375042	–	–
*Dipsaselegans*	MZUTI 3317	–	MH341021	–	MH375033	–	–	–
*Dipsasellipsifera*	MZUTI 4931	–	MH341024	–	MH375030	–	–	–
*Dipsasgaigeae*	JAC 28587	–	–	–	JX398613	JX398462	JX293850	JX398735
*Dipsasgeorgejetti*	MZUA.RE.121	–	MH341025	–	MH375024	–	–	–
*Dipsasgracilis*	MZUTI 3331	–	MH341030	–	MH374995	–	–	–
*Dipsasindica*	QCAZ 13305	MH341089	MH341037	–	MH375006	MH375043	–	–
*Dipsasjamespetersi*	QCAZ 9190	–	MH341042	–	MH375014	–	–	–
*Dipsasklebbai*	MZUTI 5412	–	MH341045	–	MH374977	–	–	–
*Dipsas* sp.	CH 8479	–	MH140683	–	–	–	–	–
	JM 663	–	MH140698	–	JX398625	JX398475	–	–
	JM 664	–	MH140697	–	JX398626	JX398476	–	–
	JM 758	–	–	–	JX398627	JX398477	–	JX398752
	JM 795	–	MH140692	–	JX398628	JX398478	–	JX398753
	SMF 97346	–	OP879850*	–	–	–	–	–
*Dipsasmikanii*	MZUSP 14658	GQ457832	GQ457771	–	KX694855	–	–	–
*Dipsasneuwiedi*	MZUSP 13972	JQ598838	JQ598898	–	–	–	–	–
*Dipsasnicholsi*	JM 812	–	–	–	JX398619	JX398469	–	–
*Dipsasoligozonata*	MZUA.RE.081	–	MH341050	–	MH375029	–	–	–
*Dipsasoreas*	MZUTI 5418	–	MH341054	–	MH374981	–	–	–
*Dipsaspakaraima*	USNM 561837	–	–	MH273777	–	–	–	–
*Dipsaspalmeri*	QCAZ 13304	MH341092	MH341065	–	MH375009	MH375046	–	–
*Dipsaspavonina*	MZUTI 4972	–	MH341068	–	MH374983	–	–	–
*Dipsasperijanensis*	UIS R-4180	–	–	–	–	OP897299*	–	–
*Dipsasperuana*	LSUMZ 1532	–	–	–	JX398622	JX398472	JX293856	JX398750
*Dipsaspratti*	MBUCV 6837	–	–	–	JX398624	JX398473	–	JX398751
*Dipsastemporalis*	MHCH 2878	–	OP879851*	OP873116*	–	–	–	–
	MHUA 14278	–	–	–	GQ334482	GQ334583	–	GQ334667
	QCAZ 5050	–	MH341069	–	MH375003	–	–	–
	SMF 97343	–	–	OP873117*	–	–	–	–
*Dipsastrinitatis*	UWIZM 2011.20.25	–	–	–	JX398629	JX398479	–	–
*Dipsasturgida*	LSUMZ 6458	JQ598839	KX660279	–	JX398696	JX398556	JX293899	JX398819
*Dipsasvagus*	KU 219121	–	KX660252	–	–	–	–	–
*Dipsasvariegata*	UTA R-15772	–	–	–	JX398601	JX398482	JX293858	JX398736
*Dipsasventrimaculata*	MCP 4870	JQ598840	JQ598900	–	–	–	–	–
*Dipsasvermiculata*	MZUTI 4738	OP839489*	OP879846*	–	–	OP897291*	–	–
	QCAZ 13563	MH341095	MH341071	–	MH374972	MH375049	–	–
	QCAZ 13582	MH341096	MH341072	–	–	MH375040	–	–
	ZSFQ 5059	OP839490*	OP879847*	–	OP897289*	OP897293*	–	–
	ZSFQ 5061	OP839491*	OP879848*	–	OP897290*	OP897292*	–	–
*Dipsasviguieri*	MHCH 2875	–	OP879852*	OP873118*	–	–	–	–
*Dipsaswelborni* sp. nov.	MZUTI 3663	–	MH341070	–	MH374989	OP897294*	–	–
	QCAZ 13825	–	MH341073	–	MH374973	MH375050	–	–
	UTA R-55939	–	–	–	JX398632	JX398483	JX293859	JX398754
*Dipsaswilliamsi*	CORBIDI 12695	–	–	–	MH374968	MH375041	–	–
*Geophisannuliferus*	JAC 27792	–	–	–	JX398699	JX398559	JX293914	–
*Geophisbicolor*	MX29-53	–	–	–	JX398637	JX398487	JX293862	JX398759
*Geophisnigrocinctus*	JAC 30704	–	–	–	JX398638	JX398488	–	–
*Geophisomiltemanus*	ENS 11496	–	–	–	JX398639	–	–	JX398760
*Geophissanniolus*	MX21-36	–	–	–	JX398692	JX398553	JX293895	JX398815
*Geophissartorii*	USNM 564144	–	–	–	JX398717	JX398585	JX293912	JX398831
*Geophistarascae*	MX28-19	–	–	–	JX398640	JX398489	JX293870	JX398761
Sibonaff.hartwegi	ICN 11463	–	–	–	–	JX398532	–	–
	ICN 11510	–	–	–	–	JX398533	–	JX398803
Sibonaff.nebulatus	CH 6614	–	–	MH140390	–	–	–	–
	UTA R-42429	–	–	–	–	JX398534	JX293887	–
	JAC 28055	–	–	–	JX398678	JX398535	JX293889	–
	JAC 28140	–	–	–	–	JX398536	JX293893	–
	JAC 28589	–	–	–	–	JX398537	JX293894	–
	JAC 30102	–	–	–	–	JX398538	–	–
	MVZ 233298	EU728583	EU728583	EU728583	EU728583	EU728583	FJ455221	FJ455189
	N068	–	–	–	JX398682	JX398542	–	JX398807
	USNM 564142	–	–	–	–	JX398547	–	JX398810
	USNM 564143	–	–	–	–	JX398548	–	JX398811
	UTA R-42431	–	–	–	JX398690	JX398549	JX293891	JX398812
*Sibonannulatus*	ADM 242	–	KX660169	–	KX660443	KX660572	–	KX651996
	ADM0007	–	KX660170	–	KX660444	KX660573	–	KX651997
	B45-57	–	–	–	–	JX398499	–	JX398770
	D167	–	–	–	JX398652	JX398501	JX293869	JX398772
	MVZ 269290	MH341097	MH341074	–	MH375034	MH375053	–	–
	N740	–	–	–	–	JX398505	–	JX398777
*Sibonanthracops*	MVZ 215680	MH341098	MH341076	–	MH375035	MH375054	–	–
*Sibonargus*	USNM 579852	–	MH140960	MH140380	JX398662	JX398511	–	JX398783
*Sibonayerbeorum*	ZSFQ 5066	OP839492*	OP879849*	–	–	OP897298*	–	–
*Sibonbevridgelyi*	MZUA.RE.424	–	–	–	MH374990	–	–	–
	MZUTI 3269	–	MH341077	–	MH374962	–	–	–
	MZUTI 5416	–	MH341078	–	MH374963	–	–	–
	RSCDSP 0391	–	–	–	JX398683	JX398543	JX293890	JX398808
*Siboncanopy* sp. nov.	JM 705	–	MH140951	–	JX398654	JX398503	–	JX398774
	JM 759	–	MH140949	–	JX398655	–	–	JX398775
	USNM 579846	–	–	–	JX398653	JX398502	–	JX398773
	USNM 579849	–	MH140947	MH140366	JX398656	JX398504	–	JX398776
*Siboncarri*	UTA R-45493	–	–	–	JX398665	JX398514	JX293876	JX398786
*Sibonirmelindicaprioae* sp. nov.	MHCH 3111	–	OP879853*	–	–	–	–	–
	MHCH 3145	–	OP879854*	OP873119*	–	–	–	–
	MHCH 3146	–	OP879855*	OP873121*	–	–	–	–
	SMF 97586	–	OP879856*	OP873120*	–	–	–	–
	SMF 97587	–	OP879857*	–	–	–	–	–
	UIS R-3515	–	–	–	–	OP897296*	–	–
	UIS R-3701	–	–	–	–	OP897297*	–	–
*Sibondimidiatus*	USNM 565824	–	–	–	JX398668	JX398517	–	JX398789
*Sibondunni*	CAMPO 533	–	MH341079	–	MH374991	–	–	–
*Sibonhartwegi*	MHUA 14511	–	–	–	GQ334556	GQ334662	GQ334579	GQ334685
	SN 0001	–	–	–	JX398684	JX398544	JX293892	JX398809
*Sibonlamari*	ASL 362	–	–	–	JX398670	JX398519	–	–
	No voucher	–	–	–	JX398671	JX398520	JX293879	JX398791
*Sibonleucomelas*	CH 5296	–	MH140972	MH140392	–	–	–	–
	CH 9135	–	MH140971	MH140391	–	–	–	–
	JM 703	–	–	–	JX398679	JX398539	–	JX398804
	JM 722	–	MH140967	–	JX398680	JX398540	–	JX398805
	MHCH 3149	–	OP879860*	–	–	–	–	–
*Sibonleucomelas*	USNM 579854	–	–	MH140393	JX398681	JX398541	–	JX398806
*Sibonlongifrenis*	MVZ 215681	MH341099	–	–	MH375036	MH375055	–	–
*Sibonmarleyae* sp. nov.	ZSFQ 5069	–	OP879861*	–	–	OP897295*	–	–
	MZUTI 3034	–	MH341075	–	MH375021	–	–	–
*Sibonnebulatus*	SN02	–	–	–	–	JX398545	–	–
	UWIZM 2011.20.26	–	–	–	JX398687	JX398551	–	–
*Sibonperissostichon*	SMF 88716	–	–	–	JX398688	JX398552	JX293888	JX398814
*Sibonvieirai* sp. nov.	DHMECN 9585	–	MH341082	–	–	–	–	–
	ENS 12500	–	–	–	–	JX398531	–	JX398802
	ENS 12459	–	–	–	–	JX398530	–	JX398801
	MZUTI 3911	–	MH341083	–	MH374964	–	–	–
	MZUTI 4810	–	MH341084	–	MH374965	–	–	–
*Tropidodipsasfasciata*	MX14	–	–	–	JX398703	–	JX293901	JX398821
*Tropidodipsasfischeri*	UTA R-38932	–	–	–	JX398707	JX398566	JX293903	JX398823
*Tropidodipsasguerreroensis*	INIRENA 2781	–	–	–	MZ287381	MZ287395	MZ287403	MZ287420
*Tropidodipsaspapavericola*	INIRENA 2805	–	–	–	MZ287392	MZ287392	MZ287400	MZ287418
*Tropidodipsasphilipii*	JAC 24811	–	–	–	JX398710	JX398570	JX293909	JX398826
*Tropidodipsastricolor*	CIG 1837	–	–	–	MZ287386	MZ287394	MZ287404	MZ287415

## ﻿Appendix 2

**Table A2.** List of PCR and sequencing primers and their respective PCR conditions (denaturation, annealing, extension and number of corresponding cycles) used in this study. All PCR protocols included an initial 3-min step at 94 °C and a final extension of 10 min at 72 °C.

Locus	Primer	Sequence (5’-3’)	Reference	PCR profile:
12S	H1557mod	GTACRCTTACCWTGTTACGACTT	Zaher et al. 2009	93 °C (1 min), 54 °C (1 min), 72 °C (2–5 min) [x25–40]
	L1091mod	CAAACTAGGATTAGATACCCTACTAT		
16S	16Sar-L	CGCCTGTTTATCAAAAACAT	Palumbi et al. (1991)	94 °C (45 sec), 53 °C (45 sec), 72 °C (1 min) [x30]
	16Sbr-H-R	CCGGTCTGAACTCAGATCACGT		
COI	RepCOI-F	TNTTMTCAACNAACCACAAAGA	Murphy et al. (2013)	94 °C (3 min), 48.5 °C (30 sec), 72 °C (1 min) [x40]
	RepCOI-R	ACTTCTGGRTGKCCAAARAATCA		
Cytb	L14910	GACCTGTGATMTGAAAACCAYCGTTGT	Burbrink et al. (2000)	94 °C (1 min), 58 °C (1 min), 72 °C (2 min) [x30–36]
	H16064	CTTTGGTTTACAAGAACAATGCTTTA		
ND4	ND4	CACCTATGACTACCAAAAGCTCATGTAGAAGC	Arévalo et al. (1994)	94 °C (25 sec), 56 or 60 °C (1 min), 72 °C (2 min) [x25–30]
	Leu	CATTACTTTTACTTGGATTTGCACCA		

## Supplementary materials

Supplementary material 1 Morphological and locality data for specimens of Dipsadini species examined, either directly, indirectly through digital photographs, or both. Codes: SVL = snout-vent length (mm); TL = tail length (mm); M = Male, F = Female. Data type Morphological https://binary.pensoft.net/file/800584This dataset is made available under the Open Database License (http://opendatacommons.org/licenses/odbl/1.0/). The Open Database License (ODbL) is a license agreement intended to allow users to freely share, modify, and use this Dataset while maintaining this same freedom for others, provided that the original source and author(s) are credited.Alejandro Arteaga, Abel Batista

Supplementary material 2 Locality data used to create distribution maps. Type localities indicated in bold type. Data type Occurrences https://binary.pensoft.net/file/800585This dataset is made available under the Open Database License (http://opendatacommons.org/licenses/odbl/1.0/). The Open Database License (ODbL) is a license agreement intended to allow users to freely share, modify, and use this Dataset while maintaining this same freedom for others, provided that the original source and author(s) are credited.Alejandro Arteaga, Abel Batista
